# *T. gondii* RP Promoters & Knockdown Reveal Molecular Pathways Associated with Proliferation and Cell-Cycle Arrest

**DOI:** 10.1371/journal.pone.0014057

**Published:** 2010-11-22

**Authors:** Samuel L. Hutson, Ernest Mui, Karen Kinsley, William H. Witola, Michael S. Behnke, Kamal El Bissati, Stephen P. Muench, Brittany Rohrman, Susan R. Liu, Robert Wollmann, Yuko Ogata, Ali Sarkeshik, John R. Yates, Rima McLeod

**Affiliations:** 1 Department of Surgery (Ophthalmology), The University of Chicago, Chicago, Illinois, United States of America; 2 Department of Veterinary Molecular Biology, Montana State University, Bozeman, Montana, United States of America; 3 Institute of Membrane and Systems Biology, University of Leeds, Leeds, United Kingdom; 4 Department of Pathology, The University of Chicago, Chicago, Illinois, United States of America; 5 Seattle Biomedical Research Institute, Seattle, Washington, United States of America; 6 Department of Chemical Physiology, Scripps Research Institute, La Jolla, California, United States of America; 7 Department of Pediatrics (Infectious Disease), The University of Chicago, Chicago, Illinois, United States of America; 8 Committees on Immunology, Molecular Medicine, and Genetics, Institute of Genomics and Systems Biology, The College, The University of Chicago, Chicago, Illinois, United States of America; INSERM U1016, Institut Cochin, France

## Abstract

Molecular pathways regulating rapid proliferation and persistence are fundamental for pathogens but are not elucidated fully in *Toxoplasma gondii.* Promoters of *T. gondii* ribosomal proteins (RPs) were analyzed by EMSAs and ChIP. One RP promoter domain, known to bind an Apetela 2, bound to nuclear extract proteins. Promoter domains appeared to associate with histone acetyl transferases. To study effects of a RP gene's regulation in *T. gondii*, mutant parasites (*Δrps13*) were engineered with integration of tetracycline repressor (TetR) response elements in a critical location in the *rps13* promoter and transfection of a yellow fluorescent-tetracycline repressor (YFP-TetR). This permitted conditional knockdown of *rps13* expression in a tightly regulated manner. *Δrps13* parasites were studied in the presence (+ATc) or absence of anhydrotetracycline (-ATc) in culture. -ATc, transcription of the *rps13* gene and expression of RPS13 protein were markedly diminished, with concomitant cessation of parasite replication. Study of *Δrps13* expressing Myc-tagged RPL22, -ATc, showed RPL22 diminished but at a slower rate. Quantitation of RNA showed diminution of 18S RNA. Depletion of RPS13 caused arrest of parasites in the G1 cell cycle phase, thereby stopping parasite proliferation. Transcriptional differences ±ATc implicate molecules likely to function in regulation of these processes. *In vitro*, -ATc, *Δrps13* persists for months and the proliferation phenotype can be rescued with ATc. *In vivo*, however, *Δrps13* could only be rescued when ATc was given simultaneously and not at any time after 1 week, even when L-NAME and ATc were administered. Immunization with *Δrps13* parasites protects mice completely against subsequent challenge with wildtype clonal Type 1 parasites, and robustly protects mice against wildtype clonal Type 2 parasites. Our results demonstrate that G1 arrest by ribosomal protein depletion is associated with persistence of *T. gondii* in a model system *in vitro* and immunization with *Δrps13* protects mice against subsequent challenge with wildtype parasites.

## Introduction

It was of interest to determine whether transcriptional regulation of a ribosomal protein (RP) in *Toxoplasma gondii* could play a role in control of this parasite's cell cycle and consequent phenotype in a model system *in vitro.* Recent studies of ribosomes and/or cell cycle [1]–[18] in a number of organisms provided precedent as they had demonstrated that RPs are critical for regulation of cell cycle, differentiation, proliferation, and responses to stresses [3], [10], [13]–[18]: For example, in *Saccharomyces cerevisiae* the absence of RPs cause cell cycle arrest in G1 [13]–[16], slowing growth, and causing differentiation; RP mutants of *Arabidopsis* [17] and *Drosophila melanogaster* “minutes” [18], have smaller numbers of ribosomes and/or severe growth impairment phenotypes. Previous studies suggested stage-associated differences in expression of *T. gondii* ribosomal proteins (RP) [19]. Furthermore, promoters of genes encoding RPs in *T. gondii* contain highly conserved *T. gondii* RP (TRPs) promoter sequences [20], [21] suggesting they could contribute to coordinated transcriptional regulation of RP synthesis. These consensus sequences of TRPs, TRP1 (TCGGCTTATATTCGG) and TRP2 ([T/C]GCATGC[G/A]), are located close to transcriptional start sites of RPs [20], [21] and are present frequently in the *T. gondii* genome. For example, a small RP (*rps13*’s) promoter has two TRP2s, in addition to a motif found to regulate bradyzoite gene expression, TGTGTG [22].

*T. gondii* also has orthologous molecules to those in other organisms that interact with transcription factors regulating RPs. Similar to other eukaryotes, activation of gene expression in *Toxoplasma* involves acetylation of histone proteins [23], [24] and other components of an epigenetic regulatory complex [23]–[26]. In the yeast, *S. cerevisiae*, MYST family histone acetyltransferase (HAT), Esa1, is recruited to RP gene promoters to regulate transcription [27]. Apetela 2 (AP2) transcription factors have been identified in *Plasmodia*, *T. gondii* and plants [28]–[38], but are not present in animals. In *Plasmodium*, a global yeast two hybrid analysis suggested that ApiAP2 proteins interact with chromatin remodeling factors and each other [39]. *P. falciparum* ApiAP2 protein PfSIP2 is key in chromosome end biology involving heterochromatin formation and epigenetic factors [40]. In the plant *Arabidopsis*, especially in stress conditions, a HAT interacts with an AP2, CBFI [41]. Thus, it seemed feasible that a similar AP2-HAT complex could play a role in regulation of transcription of RPs in *T. gondii*. Consistent with this hypothesis, the TRP2 promoter element had been reported to contain bases that bind a *Plasmodium* AP2 [29].

Little was known about which of *T. gondii*'s ribosomal components are essential for ribosomes to function properly. Thus, a new system using reagents described earlier [20], [21] was attractive for use to create a tightly regulated, conditional knockdown of a RP. To attempt to create this tight, conditional knockdown of a RP, a putative RP small subunit 13 gene (*rps13*) had been selected earlier [20], [21]. This RP was selected because it had one major transcriptional start site [20], [21]. It turned out to be a fortuitous choice because recently, other organism's RPS13s have been found to be critical for catalysis and initiation of translation. These RPS13s also are key in the association of small and large ribosomal subunits, and the point at which tRNA, mRNA and amino acids assemble to initiate protein synthesis in other organisms [4]–[8]. We considered it possible that expression of this RP gene selected earlier, if it truly were the homologue of RPS13 in other organisms, would be essential for ribosome assembly and function. Its absence would alter *T. gondii* profoundly.

The use of a transactivator system had successfully created conditional knockdowns of *T. gondii* genes [42]. A tetracycline repressor (TetR) protein system [43] for *T. gondii* had been described earlier although regulation of a functional endogenous gene product was not accomplished [20], [21]. The need for tetracycline to enter encysted bradyzoites to turn a gene off was a theoretical limitation of the current transactivator system. Another challenge for the work presented herein, was to create, validate, and characterize a tightly regulated, conditional, “tetracycline on” (called herein “+ATc”) system in *T. gondii* that would stably modify gene expression, independent of promoter strength. In studies, foundational for the present work, tetracycline repressor response element (TetO)-modified promoters for *T. gondii rps13* had been created [20], [21] demonstrating an optimal location for insertion of TetO elements by mapping the promoter to optimize tight regulation. If this putative RPS13 functioned as RPS13s in other species and was essential, it was hypothesized that a RPS13 conditional knockdown parasite could be created and characterized [20], [21].

Herein, our studies provide new empirical information concerning ribosomal protein (RPs) promoters and promoter associated proteins. A role of a *T. gondii* ribosomal protein, (and hence ribosomes) including its regulation and production, was investigated using an inducible knockdown system [20], [21]. Information concerning *T. gondii* proliferation and persistence in an *in vitro* model system was obtained. Conditional knockdown of *rps13* created a G1 arrested parasite that persisted in this tissue culture system for months. However, this mutant parasite did not appear to persist *in vivo* and protected mice against challenge with wildtype parasites.

## Results

### Modeling of the structure of the putative *T. gondii* RPS13 demonstrates that it is likely part of the ribozyme

**Figure 1:**
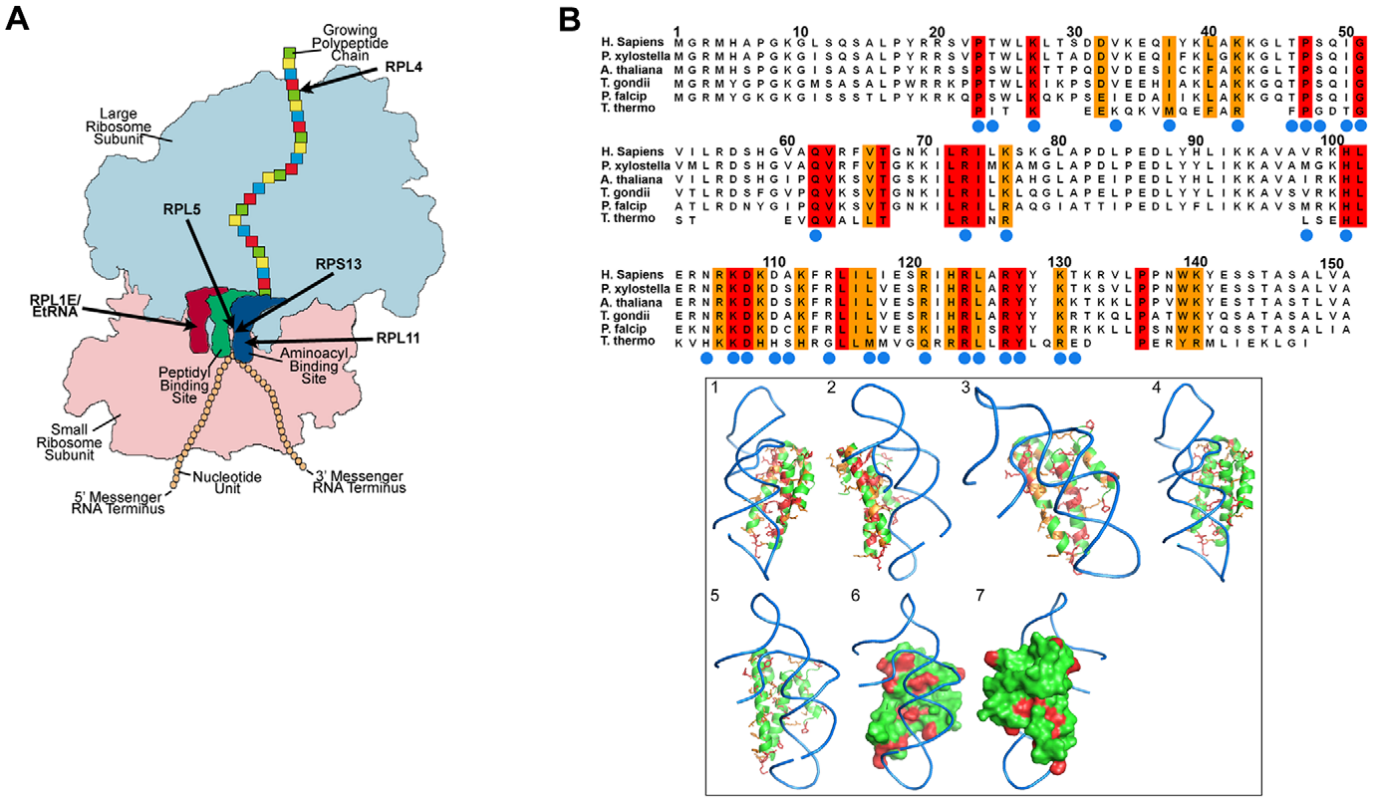
Structural model of ribosomes and RPS13 sequence alignment. (**A**) Structural model of ribosome showing key locations of critical features of ribosomes and the ribosomal proteins. These are relevant to our model and critical in ribosome function in other organisms. RPS13 (59.m03516) is in the catalytic site. RPS13 and RPL5 are contiguous. RPL5 (31.m00914) and RPL11 (583.m00014) occupy key locations and RPL4 (42.m0584) is also in a key place at the point of egress of the assembled polypeptide chain from the ribosome. Ribosomal RNA assembly protein (55.m00169) and Elongation Factor (207.m00015_x and 207.m00015) interact with the ribosome at the catalytic site. (**B**) Sequence alignment of the *T. gondii* ribosomal protein S13, with similar sequences from mammalian, plant, bacterial and parasitic sources, with the *T. thermophilus* RPS15 sequence at the bottom. Those residues that are identical and similar are boxed in red and orange, respectively. Those residues contacting the bound RNA in the RPS15 structure are highlighted by a blue circle in the sequence alignment, showing that the conservation clusters around the RNA binding site. The right panel shows a structural model of the *T. gondii* RPS13 structure, based on the *T. thermophilus* RPS15 structure which is in complex with ribosomal RNA, shown as a blue ribbon. For both the cartoon and surface representations of the RPS13 structure those residues conserved are shown in red. (See Movie S1.)

Because RPS13 was selected for conditional knockdown, and because of its key location in ribosomes of other species (Figure 1A) we wanted to determine more definitively that the putative RPS13 was likely to be a true RPS13 based on its structure. If it were a true homologue of other species RPS13s it would be likely to be essential for ribosome function. Thus, a structural model of *T. gondii* RPS13 was produced using the phyre modeling server. This model used the solved *Thermophilus* ribosomal protein RPS15 structure as a starting model. Analysis of the sequence alignment between various RPS13 proteins and the *Thermophilus* RPS15, protein from which the *T. gondii* model is based, shows a significant amount of sequence similarity between not only the RPS13 family, but the RPS13 and RPS15 proteins (Figure 1B), particularly at the C-terminus. Furthermore, the RPS15 structure is in complex with ribosomal RNA. Analysis of those residues that form the RNA binding site show strong conservation between the RPS13 and RPS15 family (blue circles in Figure 1B top panel). Moreover, the sequence conservation between the RPS13 and RPS15 family is located primarily around the RNA binding site, suggesting that the mode of RNA binding in the RPS13 and RPS15 family is similar (Figure 1B bottom panel; Movie S1). The critical location for the homologous RPS13 in the catalytic site of ribosomes of other organisms suggest that this *T. gondii* RPS13 is likely to be essential. This finding made the creation and determination of the phenotype of a conditional knockdown to characterize *T. gondii* RPS13 function of interest. There were not obvious unique features distinguishing the parasite and human RPS13s.

### Analyses of proteins bound to RP promoter elements reveal key regulatory pathways

**Figure 2:**
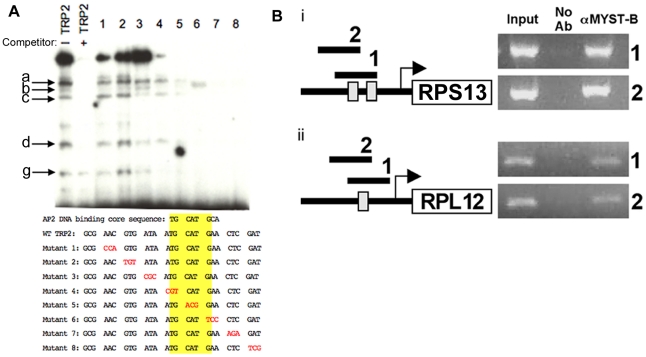
Ribosome gene promoters and transcription. (**A**) Nuclear extract proteins that associate with the TRP2 element. In EMSA with radiolabeled TRP2 probes with (+) and without (−) cold competitor TRP2 with tachyzoite nuclear extract, note mobility-shift which indicates that there is protein in the nuclear extract that binds to TRP2 which is competed by cold TRP2. Arrows with letters a to g in the TRP2 EMSAs designate some of the bands that are no longer demonstrated when cold competitor for TRP2 is present. These letters designating bands are the same as in Figure S1B. There is also a similar mobility-shift for TRP1 with cold competitor (Figures S1A and B). Arrow in Figure S1A indicates one level in an EMSA with TRP2 at which gel shift occurs for which there is no similar shift in mobility for a band in TRP1. Similar mobilities of proteins in TRP1 and TRP2 EMSAs do not necessarily indicate identical bound or associated proteins. Mapping of key bases in the TRP2 elements shows that TRP2 contains a motif TGCATG known to interact with an Apicomplexan Apetela 2 transcription factor which we find herein is critical for binding nuclear extract proteins. The loss of different bands with differing mutations in the full TRP2 promoter indicates that additional bases also contribute to binding molecules in nuclear extracts. (**B**) TgMYST B appears to associate with RP gene promoters. Tachyzoites from a stable transgenic parasite clone expressing Flag-TgMYST B were processed for ChIP using anti-Flag antibody. PCR analysis for two distinct regions of RP gene promoters RPS13 [i] and L12 are shown [ii]. They are indicated as 1 and 2, respectively. Input DNA was used as a positive control for each primer pair and a ChIP sample without antibody was included as a negative control. TRP1 and two TRP2 elements are represented as gray boxes in the promoter region of each RP gene shown.

TRP2 is present in two places in the *rps13* gene's promoter and TRP1 is present in promoters of other *rp* genes [20], [21]. This made it of interest to characterize whether TRP1 and TRP2 bound nuclear extract proteins and if so which promoter elements were critical for this binding. If they bound, further characterizing nuclear extract proteins that associated with these promoter elements also would be of interest. EMSAs using ^32^P labeled TRP1 or TRP2 promoter elements show that tachyzoite nuclear extracts cause mobility-shifts in TRP1 and TRP2, which are competitively inhibited by cold probe (Figures 2A and S2A). Nucleotide sequences within TRP2 elements of the RPS13 promoter which are critical for binding proteins were more precisely identified. This was accomplished as follows: Three base pair linker scan mutants (1 to 8 in Figure 2A) that spanned TRP2 elements were synthesized individually and radiolabeled. EMSAs were carried out using *T. gondii* nuclear extract with these radiolabeled wildtype or mutant TRP2 elements and binding pattern observed. With mutant TRP2 probes, alteration of nucleotide sequences TGCAT, a known AP2 binding motif [29] shaded yellow in Figure 2A bottom, in mutants 4 to 8 (red font, bottom panel, Figure 2A) abolishes or markedly reduces protein binding. The highest, slow-migrating complex is most intense (Figure 2A), when compared with other bound protein(s), suggesting that the upper complex contains multiple proteins. There was absence of binding with mutants 7 and 8, suggesting that there are associated proteins in addition to those that are in the complex with TGCAT.

We then studied whether *T. gondii* HATs MYST A and B associated with TRP1 and/or TRP2, because Stockinger [41] had found a HAT associated with an *Arabidopsis* AP2 and TGCAT is an AP2 binding motif. Also, LaCount [39] had found that ApiAP2s interact with chromatin remodeling factors and each other. In addition, Fluek [40], had found a major role for the *Plasmodium falciparum* ApiAP2 protein PfSIP2 in chromosome end biology. αMYST A and αMYST B antibodies each altered TRP1 or TRP2 element binding of nuclear extract in EMSAs with shift or modification of intensity of bands (Figure S1B). To confirm by another method that TgMYST HATs are associated with RP promoter regions, and more specifically the TRP elements, we also used a chromatin immunoprecipitation (ChIP) assay. ChIP was performed using αFlag antibody and parasite lysate derived from RH strain tachyzoites engineered to express a Flag-tagged form of TgMYST B. PCR for various regions of RP promoters of RPS13 and L12 was then performed on the immunoprecipitated DNA. These data indicate that Flag-TgMYST B associates with each RP promoter, including regions containing a TRP element (Figure 2B). Additional methodological details and controls are presented in Text S1. These results indicate that these TRPs in promoters of *Toxoplasma* RP genes may associate with HATs.

Mass spectrometry of proteins extracted from EMSAs of TRP1 and TRP2 bound by nuclear extracts of *T. gondii* were then performed. TRP1 and TRP2 associated nuclear extract proteins indentified by mass spectrometry are shown in Table S1. These included Ap2s, zinc fingers, zinc finger binding protein, a cold shock DNA binding protein, SWI2/SNF2, CAM kinases, GCN5, dead box helicases, NMD3, serine threonine phosphase 2C, a splicing protein, eukaryotic translation initiation factors, SnoRNA, MA3, transcription initiation factor EIF IIB, a ribosome biogenesis regulatory protein, nuclear proteins NOPs, chaperones, heat shock proteins, and RPL5.

### Creation and characterization of rps13 conditional knockdown parasites (Δrps13) *in vitro*

#### Generation of ATc controlled conditional knockdown of an endogenous rps13 gene in *T. gondii*

**Figure 3:**
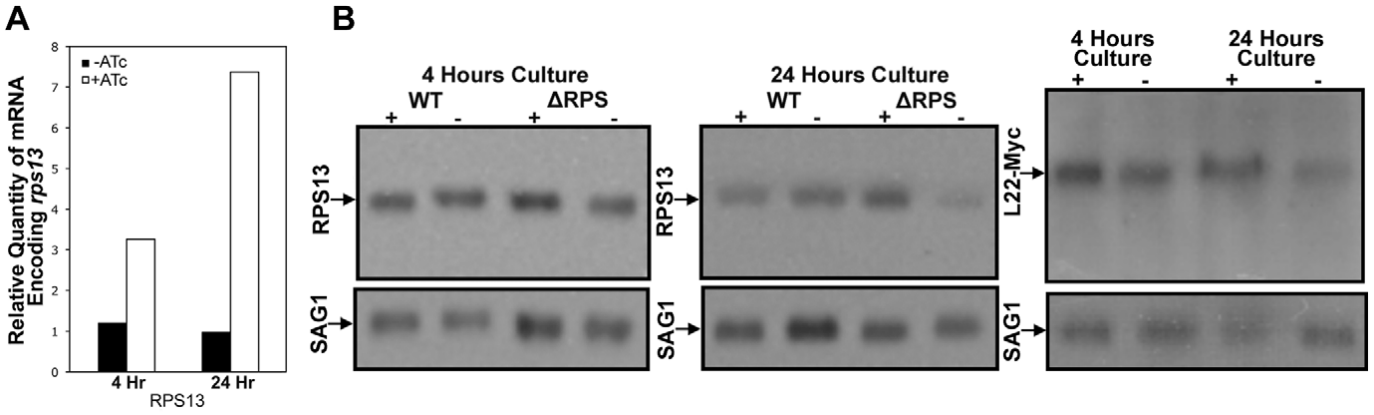
Characterization of conditional mutant parasite *in vitro*. (**A**) Quantitative real time PCR analysis of the expression of *rps13* in mutant clone 3, QRT-PCR using cDNA from parasites grown for 4 or 24 hours ±ATc shows 3-fold and 10-fold decrease in transcript levels of *rps13* at 4 hours and 24 hours of culture -ATc respectively. These results were reproducible in 2 replicate experiments. (**B**) Western blots analysis of RPS13 expression with a modest decrease at 4 hours, robust decrease at 24 hours (left panel) and marked decrease on day 3 after removing ATc (Figure S3). Western blot analysis of cMyc tagged RPL22 (right panel) shows a very slight decrease at 4 hours and a moderate decrease at 24 hours. The parallel decreases in RPS13 and RPL22 can also be seen in FA.

A targeting vector construct containing the RPS13 promoter fragment in which four TetO elements had been integrated in tandem just upstream of the transcription start site as described previously [21] was used to make conditional RPS13 knockdown mutant parasites herein (Figure S2). The targeting vector described above [21] was transfected into ΔHXGPRT RH parasites to facilitate recombination of 4 TetO elements into the endogenous RPS13 promoter following the strategy outlined in Figure S2A (top, middle and bottom). The resolved clone 1 of the mutant parasites with the RPS13 endogenous promoter bearing 4 TetO elements was transfected with a plasmid expressing a yellow fluorescent-Tet-repressor (YFP-TetR) chimeric protein [20] that would block transcription of RPS13 by binding the TetO elements in the promoter. Twelve clones were selected for expression of the YPF-TetR protein using fluorescence microscopy (data shown only for clone 3 in Figure 3A). Figure S2B shows data comparing growth of parental RH-HXGPRT and Δrps13 parasite clones 3 and 9 in the presence of anhydrotetracycline (+ATc). The Δrps13 parasites cultured with +ATc grew in fibroblasts at approximately the same rate as the wildtype RH parasites and the parental HXGPRT parasites.

#### Anhydrotetracycline regulates rps13 transcription

To determine whether there was regulation of *rps13* messenger RNA, quantitative real-time PCR was used to measure *rps13* transcription in parasites with and without anhydrotetracycline (±ATc). RNA samples were taken from clone 3 grown for 4 and 24 hours. Amount of measured *rps13* was normalized to total RNA. Primers at the C terminus outside the region of the construct for homologous recombinants were used. The effects of culturing the conditional knockdown parasites -ATc on abrogating *rps13* mRNA were apparent early, at the 4-hour time point. Reduction of mRNA was robust by 24 hours. We found a 3-fold diminution of mRNA at 4 hours and a 10-fold diminution at 24 hours (Figure 3A). Abnormal transcription of *rps13* within hours of removing ATc, documents for the first time the utility of this new knockdown system.

#### Anhydrotetracycline regulates expression of RPS13 and proliferation of rps13 conditional knockdown parasites

**Figure 4:**
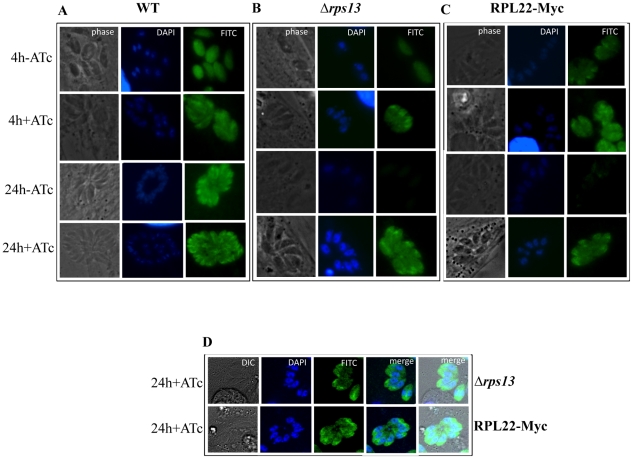
Immunofluorescence assay analyses of RPS13 and RPL22. (**A–C**) Immunofluorescence assay analysis of the expression of RPS13 in wildtype (wt,A) and *Δrps13* (B) parasites at 4 and 24 hours of culture ±ATc. RPS13 protein is shown as green fluorescent protein with the parasite and host cell nuclei stained blue. In *Δrps13* parasites, in the absence of ATc, RPS13 expression is shown to decrease modestly at 4 hours and significantly at 24 hours of culture. Rabbit αRPS13 was used as described in the text. Immunofluorescence assay analysis of the expression of Myc-tagged ribosomal L22 protein (L22-Myc) in L22-Myc translateds *Δrps13* mutant parasites at 4 and 24 hours of culture ±ATc (C). L22-Myc protein is shown as green fluorescent protein with the parasite and host cell nuclei stained blue. In the absence of ATc, L22-Myc expression is shown to decrease modestly at 4 hours and significantly but slightly less than RPS 13, at 24 hours and 4 days (Figure S3) of culture (**D**) Confocal microscopy shows RPS 13 and RPL22-Myc at the perimeter of the nucleus in the area of the endoplasmic reticulum and golgi apparatus in the merged image.

It was not known whether *T. gondii* RPS13 would be essential for ribosome stability and biogenesis in this parasite, or whether there would be regulation of or effect on replication of *T. gondii* with a four TetO construct. Therefore, clones 3 and 9 were selected for further examination. Western blots, using clone 3, at 4, 24 and 48 hours after removing ATc were performed using αRPS13. Western blots showed modest diminution of RPS13 at 4 hours and a marked diminution of RPS13 at 24 and 48 hours in the absence of ATc (Figures 3B and S3A). Consistent results were obtained from immunofluorescence assays with αRPS13 (Figures 4 and S3B).

**Figure 5:**
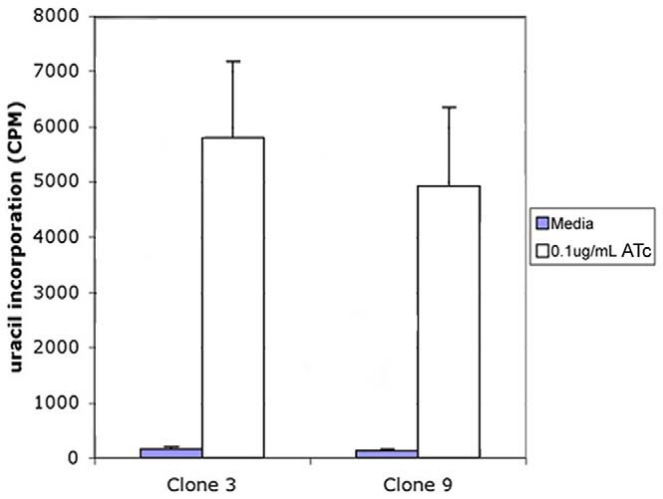
Uracil incorporation assay. Parasites were grown for 3 days ±ATc at which point radiolabeled uracil was added. Uracil incorporation into parasites was measured 24 hours later. Both clones 3 and 9 showed a 97% decrease in incorporation of uracil at 4 days.

Proliferation rates ±ATc were studied by visual inspection of live cultures and by measuring radiolabeled uracil uptake. -ATc, uracil uptake decreased by over 97% in each clone at 4 days (*P*<0.001) (Figure 5). No multiplying *T. gondii* or lysis of monolayers by replicating organisms in ATc-deprived cultures were observed by light microscopy.

#### Diminution and then absence of the expression of RPL22-Myc in mutant parasites in absence of ATc

To further characterize the effect of knockdown of RPS13 on expression and localization of large ribosomal proteins and assembly of large subunits of ribosomes, stable transfectant parasites expressing RPL22-Myc were created as described [44], [45]. These parasites were cultured ±ATc for 4 hours, 24 hours, or 4 days and parasitized fibroblasts were harvested and processed for immunofluoresence assays using αcMyc and αSAG1. While Myc-tagged ribosomal protein was abundantly expressed and was ubiquitously distributed in mutant parasites expressing RPS13, the same protein was scantily expressed in parasites with knocked-down RPS13 (Figure 4B). RPL22 diminished slightly at 4 hours, with a significant decrease being observed at 24 hours that was greater at 4 days (Figure 4C, S3B). RPS13 and RPL22 were in the perinuclear area of endoplasmic reticulum and golgi (Figure 4D). Western blot results for RPS13 and RPL22 expression corroborated the immunofluorescence assay observations (Figure 3B). Diminution of RPL22 occurred a little more slowly than RPS13. These studies demonstrate the importance of RPS13 for synthesis of RPL22 and biogenesis and assembly of ribosomes. Thus, diminished synthesis of additional RPs likely enhances the effect of knockdown of *rps13* on ribosomes.

#### Stability of mutant parasite phenotype in 40+ passages in tissue culture

To determine the stability of the RPS13 conditional knockdown system in the mutant parasites under prolonged *in vitro* culture, the mutant parasites that had been continuously cultured for over 40 passages in the presence of ATc were then cultured in the presence or absence of ATc. In the presence of ATc, the mutant parasites maintained a proliferative phenotype. In the absence of ATc, proliferating parasites were not noted in the cultures examined using an inverted microscope to monitor tissue cultures. The phenotype persisted in parasites that had been passaged many more than 40 times in the presence of ATc. These results indicate that the mutant parasites are stable *in vitro* even over prolonged continuous culture conditions and do not revert to the wildtype genotype.

#### Attenuated parasites can be rescued at 1 month and 2.5 months in tissue culture

To determine whether conditional knockdown of *rps13* was lethal, mutant parasites (clone 3) with conditional knockdown of *rps13* (-ATc) were cultured in multiple experiments for times up to 75 days during which time no lytic plaques or obviously replicating parasites could be observed microscopically. After the varying periods of culture -ATc, the infected fibroblasts were scraped off and aliquots of the cell suspension split into two equal parts and used to infect fresh fibroblasts ±ATc. In the fibroblast cultures +ATc, rapidly proliferating parasites could be observed as early by approximately 5 days for parasites previously maintained for 27 and 75 days -ATc. These parasites went on to destroy fibroblast monolayers in the presence of ATc. In contrast, parasites in -ATc, still did not proliferate and did not destroy the monolayers. There were two replicate trials performed to 2.5 months. By 5 days after addition of ATc there were replicating parasites in these cultures (Data not shown).

#### Conditional knockdown of RPS13 induces changes in the expression patterns of SAG1 and BAG1

**Figure 6:**
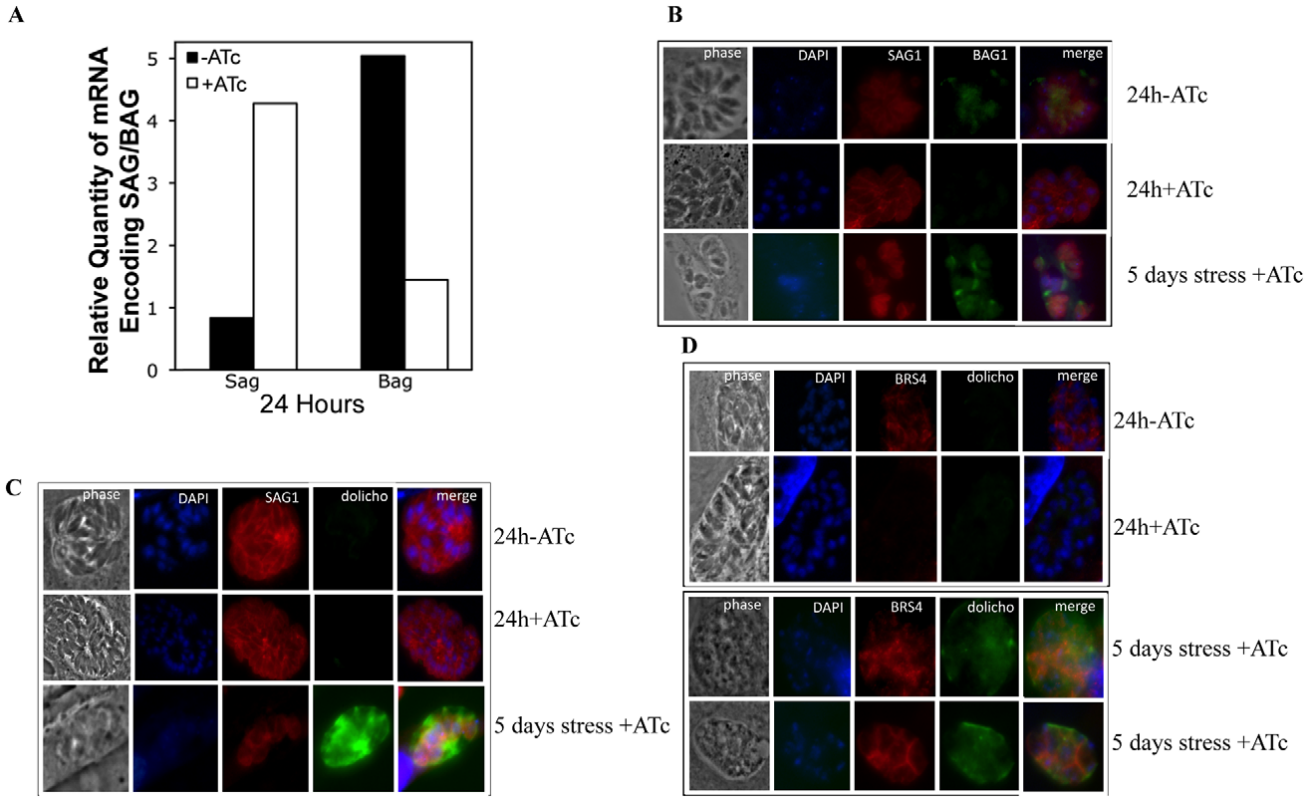
Message for SAG1 and BAG1 ±ATc for 4 or 24 hours of culture and immunofluorescence analyses for BAG1, SAG1, SRS9, and Dolichos. (A) Increased message for BAG1 and decreased message for SAG1 -ATc at 24 hours. There was no effect on BAG1 and SAG1 mRNA at 4 hours. These results were reproducible in two replicate experiments. WT =  wildtype strain. RPS13 =  conditional mutant. (B) To visualize the parasite cysts, Dolichos staining is green. Tachyzoite surface protein, SAG1 staining is red. The parasite and fibroblast nuclei stain blue. In either + or – ATc conditions, there is no Dolichos staining. In C, SAG1 staining is green, BAG1 staining is also green, DAPI is stained blue. -ATc BAG1 is present while +ATc BAG1 is absent. Control staining with 5 days of pH stress +ATc also is shown. (D) –Atc for 24 hours BRS4/SRS9 staining (red) is seen in some parasites but Dolichos staining is absent (top). Control staining of +ATc parasites that have been stressed with pH shock (bottom panel) show both green Dolichos staining and red BRS4/SRS9 staining, with dapi marking the parasite nuclei. –ATc parasite BAG1 transcript and protein increase. BRS4/SRS9 protein increases but Dolichos is not present in a cyst wall. These *Δrps13* parasites when stressed with pH shock are capable of differentiating to parasites that form structures that stain with Dolichos as well as producing BAG1 and BRS4. -ATc, some *Δrps13* parasites stain for BAG1 and SRS9, but not for Dolichos.

The prolonged persistence of the dormant parasites in culture suggested a bradyzoite-like phenotype and it seemed possible that knockdown of *rps13* might initiate a stress response. Thus, real-time PCR analysis of the transcript levels of stage specific markers for tachyzoites (SAG1) and bradyzoites (BAG1) in clone 3 cultured ±ATc for 4 hours, 24 hours and 5 days was performed. At 4 hours of culture, there was no change in SAG1 expression while there was a notable increase in BAG1 transcript levels. By 24 hours of culture BAG1 transcripts had increased substantially while that of SAG1 had decreased. The dynamics of both SAG1 and BAG1 transcript levels suggested a coordinated effect of perturbation of gene expression. The ratio of BAG1 to SAG1 was elevated in the absence of ATc suggesting an early “stress” response. These findings were reproducible in two replicate experiments (Figure 6A). Immunostaining for SAG1 and BAG1 confirmed these findings (Figure 6B, C). Staining with αBAG1 and αSRS9/BRS4 (another bradyzoite protein) were present in some parasites (Figure 6B, D top), but reactivity with Dolichos was absent (Figure 6C top, D top). In stressed parasites used as controls for this staining, BAG1 (Figure 6B) and BRS4/SRS9 (Figure 6C, D bottom) were also present. Dolichos staining of a cyst wall component was also present (Figure 6C bottom, D bottom).

#### Parasites are arrested at G1

**Figure 7:**
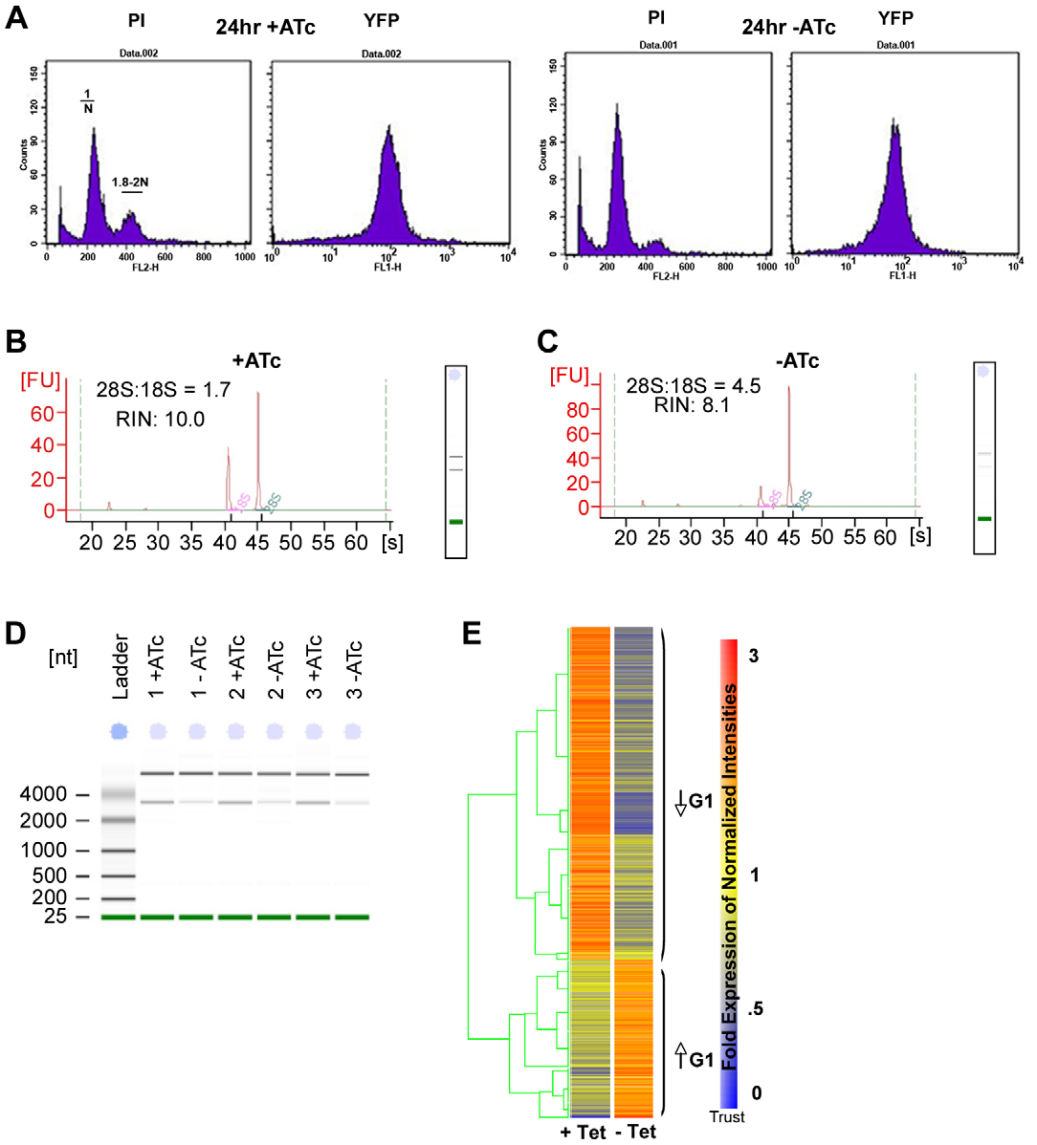
Arrest in G1 and transcriptional analysis of *Δrps13* ±ATc. (**A**) G1 arrest shown by FACS. There is an increase of 1N and decrease of 1.8 to 2N population at 24 hours -ATc, a profile seen at the G1 phase of synchronously growing parasites. (**B** to **D**) Reduction of small subunit 18S rRNA. RNA samples used for microarray hybridizations were analyzed on a 2100 Bioanalyzer to determine RNA integrity. (**B,D**) +ATc samples exhibit normal rRNA ratios (1.7–2), demonstrating robust, intact RNA. (**C,D**) -ATc samples exhibit unusually high rRNA ratios (4.5–4.8) that were out of the normal range, suggesting abnormal rRNA integrity. The decreased area of 18S rRNA peaks in the -ATc samples suggest 18S breakdown. Panel C shows 3 replicates indicated by “1, 2, 3.” (**E**) Transcriptome analysis of *Δrps13* ±ATc parasites. Transcriptional differences demonstrate arrest in G1 of the cell cycle for -ATc parasites. Over 60% of the genes that have diminished expression -ATc overlap with genes that are diminished at G1, and over 40% of genes with increased expression -ATc overlap with genes that are up regulated at G1. Annotated transcriptome data are included in Table S2.

Abrogation of ribosomal proteins in yeast results in arrest in G1 [13]–[15]. Thus, it was of interest to determine whether abrogating ribosome function might cause arrest in G1 of the cell cycle. Tachyzoites cultured +ATc exhibit a normal asynchronous profile but those cultured -ATc are largely 1N at 24 hours as demonstrated by staining with propidium iodide and subsequent FACs analysis (Figure 7A). The 2N peak in -ATc samples is diminished compared to the peak in +ATc samples. By gating plots, percentage of parasites in G1 was quantified as 68.5% +ATc, a G1 profile seen in asynchronously growing parasites [46], [47], and as 85% -ATc as seen in synchronously growing parasites. Therefore, by FACS, -ATc parasites show an increased G1 distribution. These studies were replicated twice and the results, 85% -ATc, 68.5% +ATc were similar.

#### Global transcriptome analysis indicates that depletion of rps13 leads to cell cycle arrest in G1

Since FACS analysis suggested that parasites were arrested in G1, transcriptomes were analyzed to determine whether RPS13 depletion would have an effect on global mRNA profile. Electrophoretic analysis of RNA used for microarray hybridization showed that -ATC, small subunit RNA (18S) is markedly diminished in RPS13 conditional mutants, but large subunit RNA is not (28S) (Figure 7B to D). Ribosomal RNA makes up >80% of total RNA in the cell, therefore rRNA quality and quantity reflect that of the mRNA population. Since the majority of the sample RNA consists of 28S and 18S rRNA species, the 28S:18S rRNA ratio has traditionally been viewed as the primary indicator of RNA quality, with a ratio of 1.7 to 2.0 considered to indicate high-quality, intact RNA. +ATc samples had rRNA ratios of 1.7, demonstrating robust, intact RNA. -ATc samples had unusually high rRNA ratios (4.5 to 4.8) that were out of the normal 1.7 to 2.0 range, suggesting abnormal RNA integrity. Decreased area of 18S rRNA peaks in these samples suggest small subunit (18S) breakdown.

**Figure 8:**
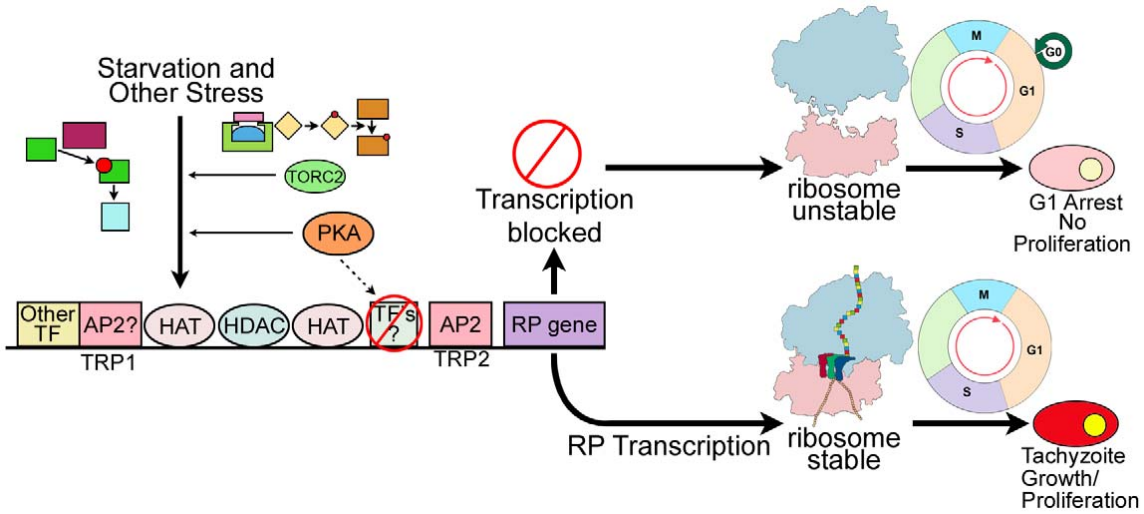
Model of interactions of ribosomal protein promoter elements, regulation of RP synthesis and G1 arrest. This model is based on data herein, which complements and is consonant with data in the literature [24]–[26], [49], [52]–[57], [73] and [Kim et al, personal communication, 2010]. This image shows a model of interactions of ribosomal protein promoter elements with complexes that regulate their transcription. It also shows the effects of diminution of transcription of a RP and the additional stress of abrogation of protein synthesis in our *in vitro* model system. In this model, stress activates signaling pathways. Signaling pathways are indicated by small colored symbols. This signaling decreases transcription of RPs. This decreased RP leads to diminution of ribosomes and arrest of proliferation, and arrest in G1. We hypothesize the following explains our findings: Stress is detected and there is signaling via PKA [24] and TOR [24], [26] which leads to diminished transcription of certain ribosomal genes. Transcription factors interacting with RP gene promoter elements such as TRP1 and TRP2 include AP2s (Kim et al, personal communication, 2010), and the response elements associate with chromatin remodelers as demonstrated herein. In this model, we propose than when there is incomplete synthesis of ribosomal proteins, then p53 is not degraded and there is cell cycle arrest in G1, as we found herein when *rps13* transcription was diminished.

Affymetrix *Toxoplasma* microarray data was analyzed to determine whether there was an association with a G1 transcript profile. Four hundred and seventy-five genes with significant gene expression differences (Welch T-test, *P* = 0.01) between ±ATc samples were identified. These gene expression differences are represented in the heat map in Figure 7E. Genes were clustered into those increased (153) or decreased (322) -ATc. There are a number of genes with diminished expression and pronounced over expression -ATc including LDH1, 4 zinc finger proteins, leucine rich protein, ABC transporter-like molecules, a bradyzoite small heat shock protein, myosin related molecules with many fold increases and one AP2 protein with a small increase (Table S2). These gene sets have a large overlap with G1 genes defined in an ongoing cell cycle expression study in *T. gondii* (Behnke, White, in preparation). Over 60% of the genes that have diminished expression -ATc overlap with genes that are diminished at G1, and over 40% of genes with increased expression -ATc overlap with genes that are up-regulated at G1. A hypergeometric distribution of this association suggests that the probability of this happening by chance is essentially zero. Thus, by both FACS and microarray analysis, parasites -ATc display a G1 profile. Figure 8 shows this G1 arrest and its consequences schematically.

### Characterization of RPS13 mutant parasites *in vivo*

#### RPS13 conditional parasites are not lethal for mice -ATc and are lethal +ATc administered on the day of infection but not at any time after one week

**Figure 9:**
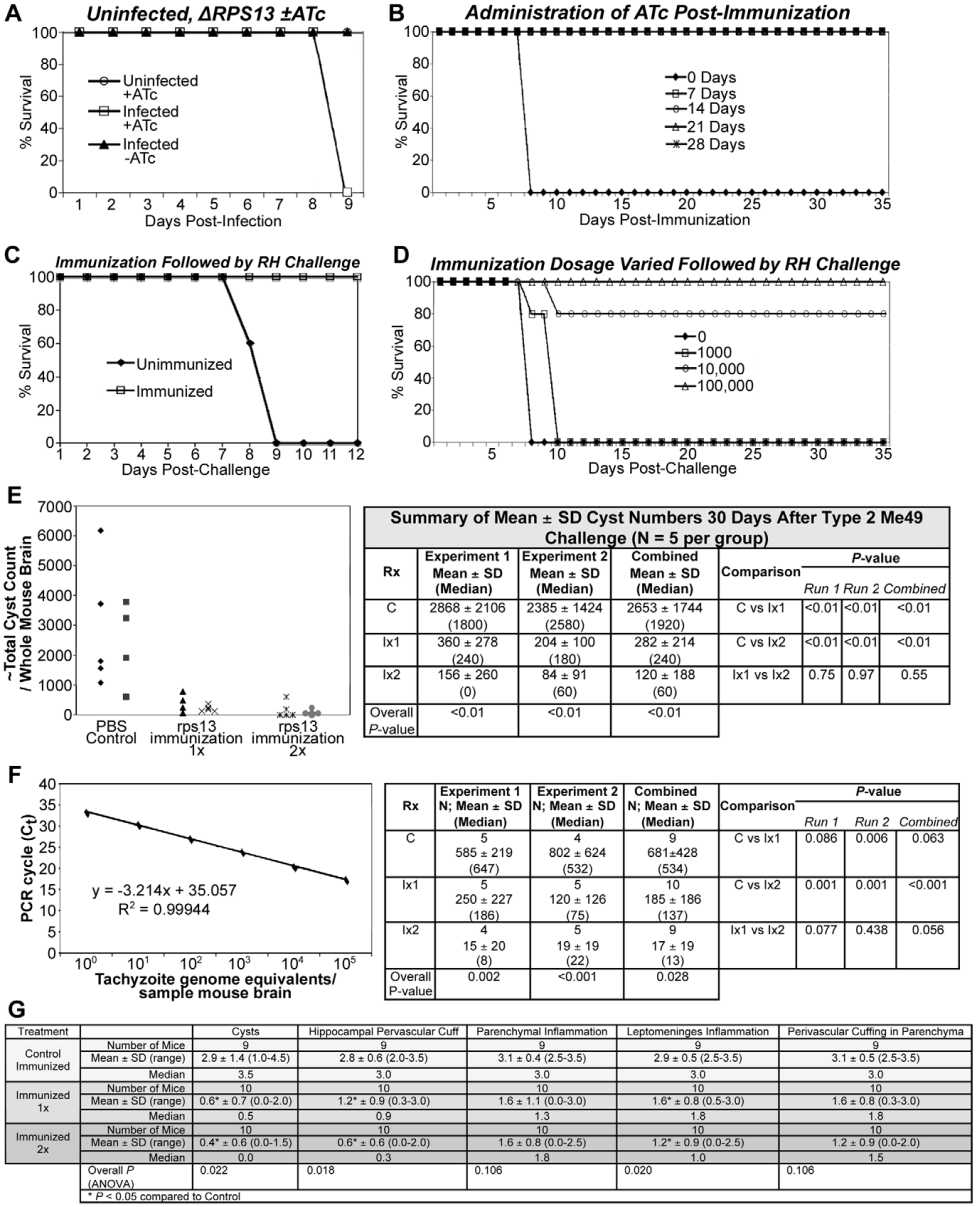
*In vivo* experiments. (**A**) Mice given RPS13 mutant -ATc survive. Data shown to day 9 but experiment continued to 6 months. Mice given RPS13 mutant +ATc simultaneously all die. Those given mutant but no ATc all survived. Two hundred RPS13 mutant parasites were administered i.p. (**B**) Administration of ATc at varying times simultaneously with or after 100,000 RPS13 mutant parasites. Simultaneous administration of ATc and parasites is lethal but not administration of ATc at 1 or more weeks after administration of 100,000 RPS13 mutant parasites. (**C**) Complete protection against challenge with 2,000 RH tachyzoites by immunization. There was complete protection with a single dose of 100,000 parasites. Challenge was 8 weeks after the immunizing dose (to 3 months). (**D**) Challenge of *Δrps13* immunized SW mice that received varying immunizing doses of *Δrps13*. Doses range from 1,000 to 100,000 *Δrps13* tachyzoites with RH strain tachyzoites. There is one hundred percent survival with 100,000 immunizing dose. (**E**) Reduction of cyst numbers following challenge of *Δrps13* immunized mice with Type 2 parasites. Immunization results in reductions in cyst numbers. (**F**) Reduction of parasite burden following challenge of *Δrps13* immunized mice with Type 2 parasites. Parasite burden demonstrated by qPCR for the 300 copy 529 base pair gene is diminished by immunization. (**G**) Reduction of brain pathology following challenge of *Δrps13* immunized mice with Type 2 parasites. Histopathology, and brain pathology following challenge of control unimmunized and mice immunized one or two times and challenged with ME49 (Type 2) parasites was analyzed. There was significantly less parasite burden and pathology following this Type 2 challenge of the immunized mice, but infection and pathology were not eliminated completely. There appeared to be somewhat greater protection following two immunizations but the differences did not reach statistical significance. Cyst number was quantitated on a scale of 0 to 5, as were hippocampal perivascular cuffing, intraparenchymal inflammatory process, inflammation in the leptomeninges, and inflammatory process in the vasculature within the brain parenchyma. Data are expressed as number of mice, mean with standard deviation, median, and range. Differences between immunized and unimmunized mice were statistically significant for all but parenchymal inflammation and perivascular cuffing in parenchyma.

The fate of these G1 arrested parasites and whether they could be demonstrated to survive *in vivo* or would be eliminated by a competent host immune response was of interest. Thus, 100 tachyzoites of the conditional mutant clone 3 were administered intraperitoneally to female Swiss Webster mice, half of which were given 0.2 mg/mL ATc to drink in their water and half of which were given water -ATc. A third group was left uninfected but given ATc to drink in their water. By 10 days, all infected mice given ATc had died, whereas all other mice were alive and remained alive for up to a year (Figure 9A). These surviving mice were treated for 10 days beginning 30 days after infection +ATc and L-NAME. L-NAME inhibits production of iNOS thereby affecting the major mediator of protection, interferon-γ [48]. All mice treated after 1 week +ATc and/or L-NAME survived (results not shown).

#### No Δrps13 parasites can be demonstrated to persist in mice

To determine whether parasites would persist and could be rescued *in vivo* by re-addition of ATc, mice received a higher dose, 100,000 *rps13* mutant parasites, and at 0, 7,14, 21, and 28 days mice were treated for 1 month +ATc. The group of mice that received treatment +ATc on day 0 died on day 9 after infection (Figure 2). However, if ATc was begun after 1, 2, 3 or 4 weeks all mice survived (Figure 9B). Even when L-NAME was added to drinking water in addition to ATc no mice died or became ill. L-NAME inhibits iNOS [48], which results in increased proliferation of wildtype parasites.

#### Robust protection against challenge with wildtype RH strain tachyzoites following immunization with Δrsp13 demonstrates that ΔRPS13 can function as a vaccine

Since *Δrps13* mutant parasites were markedly attenuated, we hypothesized that they therefore could be immunogenic, presenting both tachyzoite and some stress related bradyzoite antigens (e.g. BAG 1), and persist at least for a short period of time while they elicited an immune response. Thus, in two replicate experiments, groups of mice were given 100,000 RPS13 conditional mutant parasites and all mice survived. They then received booster doses of 100,000 RPS13 conditional mutant parasites 2 weeks and 4 weeks later without ill effect. Two weeks later they were challenged with 2,000 RH tachyzoites and all unimmunized control mice died while all immunized mice survived (Figure 9C). In separate experiments (data not shown) immunized mice challenged 1 year later were protected (∼80 to 90% survival) against otherwise lethal challenge.

A similar set of two replicate experiments was performed after immunization with a single dose of 1,000 or 10,000 or 100,000 mutant parasites with challenge with 2,000 RH strain parasites 8 weeks later. A dose of 100,000 parasites resulted in complete protection. Doses of 10,000 parasites resulted in ∼50 to 80% protection and lower doses were not protective (Figure 9D).

To determine whether any parasite DNA could be found to persist in brains of these immunized and challenged mice, PCR with primers to detect the multi-copy B1 gene and quantitative PCR to detect the 300 repeat copy 529 base pair gene was performed with brain from mice infected for prolonged periods of time after immunization with *Δrps13* -ATc. This did not reveal *T. gondii* B1 or 300 copy (529 bp) gene DNA in any of 8 mice (data not shown). RH tachyzoite DNA (this PCR method was sensitive in detection of 4 parasites) was the positive control for this quantitative PCR experiment (r^2^ = 0.99).

#### Protection against Type II parasites

In these experiments, there were 5 unimmunized mice in each trial with a total of 10 unimmunized mice. There were 5 mice immunized one time (1×) and 5 mice immunized two times (2×) mice in each trial and thus a total of 10 immunized mice for each type of immunization (1×, 2×). They were challenged with 50 Me49 cysts i.p. and at 30 days after challenge, cyst numbers were found to decrease markedly in immunized mice (Figure 9E; *P*<0.01). In these experiments, qPCR of brain using the 300 copy 529 bp gene was performed following challenge of control and immunized mice. This demonstrated marked diminution in parasite burden in brain following immunization, most dramatically for those immunized two times compared to the unimmunized group (Figure 9F, *P*≤0.001). Pathology still was present, although less, and rare cysts were seen following immunization and challenge when compared with unimmunized controls. Pathology was more focal in the immunized mice but quantitation of histopathology did not differ significantly between mice in groups that had been immunized once or with a booster immunization (2×) (Figure 9G).

## Discussion

Our finding that abrogation of a ribosomal protein causes a specific G1 arrest confirms the strength of the refined Tet model for conditional gene knockdown. This influence of ribosomes on proliferation and persistence has not been demonstrated previously in *T. gondii,* although there is precedent for this fundamental biological process in other eukaryotes such as yeast. Our findings provide an *in vitro* model which suggests that *T. gondii's* sensing of intra-parasite stresses could modulate regulation of transcription of its *rp* genes. RPs influence biogenesis of ribosomes, and subsequently, the ability of the parasite to either proliferate or arrest in G1. Analysis of promoters of RPs led Schaap and van Poppel to postulate that two conserved *rp* gene promoter cis elements, TRP1 and TRP2, regulate expression of *rp* genes, and thereby, biogenesis of ribosomes [49]. Schaap and van Poppel had postulated that TRP2 would be an enhancer and TRP1 a suppressor of *rp* transcription [49]. Empirical evidence from our studies herein, along with the work of others (Kim et al, personal communication, 2010, [50], [72]) is consistent with a role for AP2 transcription factors that interact with TRPs and regulate RP synthesis, and thus specify parasite cell and, at least in part, life cycle stages. Using rolling mutations to study TRP2, we found that a specific six base TRP2 motif is essential for binding nuclear extract proteins (Figure 2). Very recently, this same TRP2 promoter element was predicted to bind an AP2 [29], and later observed to bind a *T. gondii* AP2, X1-3 (K. Kim, personal communication, 2010). Movement of AP2s to the nucleus occurs in waves associated with the cell cycle and transcription of genes [72]. In G1a of the cell cycle in the tachyzoites, hundreds of genes including ribosomal genes are expressed. These have the TGCATG in their promoters [72]. Another group of investigators found that the TGCATG element in TRP2 in the promoter of *rpl9* enhances transcription of a reporter gene [50] in *T. gondii*, as van Poppel and Schaap had postulated. These latter studies occurred while we found that these six bases that are part of the TRP2 promoter element were necessary, but not sufficient, for binding the nucleoproteins in the complex in our EMSAs. We found that additional C terminal bases also were critical for binding/associating with nuclear extract proteins. The loss of different bands with differing mutations in the full TRP2 promoter element suggests that additional bases also contribute to a complex or complexes of associated nuclear extract proteins. For mutants 7 and 8 all associations with all nuclear extract proteins are abrogated. One explanation for this might be that an association with some of the other proteins with bases absent in mutants 7 and 8 could be critically dependent for or on protein(s) associated with TGCATG. Otherwise, it might be expected that only some of the bands would disappear because the TGCATG core site is intact in mutants 7 and 8. Recent work of others [51] indicates approaches whereby transcription factors such as AP2s might be inhibited that may be pertinent to future work. Future studies of mutations in the *rps13* promoter using a luciferase reporter may be useful in clarifying the importance of these bases in both of its TRP2 elements in enhancing transcription. Their relative importance and interaction with the TGTGTG promoter element, previously shown to be important in bradyzoite gene transcription, in the *rps13* promoter also will be of interest in future studies.

In addition, we found that αMYST A and B caused shifts in our EMSAs with both TRP1 and TRP2. Myst B (GCN5) was identified by mass spectrometry of nuclear factors binding TRPs 1 and 2. Also, in ChIPs, antibody to flag tagged MYST B suggested interactions with TRP1 and 2. Attempts to perform this ChIP study for TgMYST A were complicated by the fact that an additional copy of TgMYST A is lethal to *Toxoplasma* [25]. These findings all support association of MYST A and B HATs with proteins bound to TRP1 and 2 or direct binding of these HATs to *rp* promoters. These results are similar to the association of HATS with AP2 transcription factors that regulate genes in *Arabidopsis* [29] and *Plasmodia* [39], [40]. A protein interaction network in *Plasmodia* demonstrated an AP2 interaction with GCN5 histone acetyl transferases and Swi2/Snf2 ATPases [39]. These MYST family HATs may bind via an AP2 complex as occurs in *Arabidopsis* [29] or could bind directly to DNA as occurs in a mammalian MYST HAT. Another histone interacting protein, HDAC3, also associated with an AP2 (TgCRC35) [23]. It is likely that other transcription factors also participate in regulation of production of RPS13 and other RPs. For example, there is also a TGTGTG in the RPS13 promoter. This motif is critical in controlling expression of genes in parasites stressed *in vitro* [22]. Proteins bound in our EMSAs (Table S1) identified by mass spectrometry suggest that certain proteins including AP2, GCN5 (MystB), Swi2/Snf2 ATPases may be in the complex in *T. gondii*. Further, future studies with knockdown and tagging these genes, ChIP, and immunolocalization will be useful in definitely proving there is truly an association and if so in characterizing the association.

We note, relevant to our model (Figure 8), as suggested by our data, that in yeast, TOR and PKA pathways regulate ribosomal genes in response to conditions of amino acid deprivation and other cellular stress-associated signals. Sensing of stress is through long chain fatty acids, ceramide synthases and ceramide phosphatases. All the molecules in the PKA pathway [53] and some in the TOR pathway [26] are present in *T. gondii* and a TOR-like protein appears to be transported into the nucleus. Putative homologues of kinases, transcription factors, cyclins and a ceramide activated protein phosphatase that is involved in sensing cellular stress and mediates G1 arrest via TORC2 in yeast have been identified in *T. gondii* [13]–[15], [54] (K. Kinsley, E. Mui, R. McLeod, unpublished data; Figure 8). The same families of proteins that interact with components of the TOR and PKA pathways in yeast (e.g. HATs) also associate with TRP2 in the promoter of *rps13* and TRP1 in the promoters of other RPs in *T. gondii.* This further suggests that TOR and PKA pathways may participate in regulation of proliferation and persistence in *T. gondii*. Roles for long chain fatty acids and ceramide synthases producing ceramides and signaling through TORC2 [55] are likely because homologues of these proteins key in stress responses in yeast also are present in *T. gondii.* Sensing of cellular stress in plants is mediated by PYR/PYL/RCAR [56] and abscisic acid which modulates a PP2 phosphatase via the ligand (ABA)-bound receptor. These pathways modulate calcineurin and calcium fluxes and have been found in *T. gondii* [57].

Further, growth arrest in yeast and animals can be mediated by depletion of ribosomal proteins. In yeast, ribosome biogenesis is linked to G1 of the cell cycle [14]. Recently Ferreiria-Cerca, Fumagalli, and others [1]–[3], [10] found that eukaryotic ribosomal proteins and subunits are delicately balanced. In this work with hepatic cells, Fumagalli found that when RPL11 and MDM2 (a ubiquitin ligase that degrades p53) interaction is modified, by diminishing RPL11, p53 degradation is altered. When p53 is not degraded, it causes cell cycle arrest in G1 [3]. Whether this precise molecular mechanism also occurs in *T. gondii* and whether it is operative *in vivo* remains to be determined. *Plasmodia* monitor nutrient support for translation of proteins before committing to DNA synthesis. The cell cycle mechanisms sensing ribosomes or translation factors [73], [74] are connected with induction of genes required for DNA replication in late G1/S through G1 cyclin regulation, although protein interactions in yeast and metazoans differ [72].

To study effects of ribosomal protein S13 on proliferation and persistence in *T. gondii in vitro*, a new conditional knockdown system was used with a TetR knockdown system using constructs discussed earlier [20], [21]. Theoretical strengths of this YFP-TetR conditional knockdown system are that the modified and the native promoter have the same robustness and also, theoretically that ATc does not need to access encysted bradyzoites for knockdown. Similar YFP-TetR conditional knockdown systems also had demonstrated stringent regulation of gene function with low concentrations of ATc in other organisms. This system had a 88-fold regulation of transcription of *rps13* using a reporter gene in *T. gondii* [20]. We subsequently, herein, successfully did find this system to be effective for conditional knockdown of the *T. gondii* endogenous RPS13 gene. Separately we have found this system is effective in regulating a novel dense granule protein and parasite enzymes (W. Witola, K. El Bissati, R. McLeod, manuscripts in preparation, 2010).

The putative RPS13 selected to attempt to interfere with ribosome function using this new conditional knockdown system was chosen because it has a single transcription start site [20], [21]. Although it was not known at the time the reagents were created [21] or our studies were initiated, this turned out to be a fortuitous choice because recently RPS13 was determined to be in the ribosome catalytic site [5]–[8], [58]–[60] in other organisms (Figure 1A). Herein, our deduced structure of the putative RPS13 selected for conditional knockdown was modeled using the published structure of *Thermophilus* RPS15 as a basis because this latter structure had been solved. This confirmed that this *T. gondii* molecule has all the features to be a RPS13. It has residues likely to interact with ribosomal RNA (blue circles, Figure 1B, on line movie RP link) and thus to function in catalysis and initiating translation rather than only as ribosome scaffold. It thereby should be critical for protein assembly. In *S. cerevisiae* the RPS13 homologue is in the rRNA/r-protein neighborhood [4]–[8] and in *E. coli* RPS13 is essential for ratcheting small and large ribosomes contiguous to each other and is the direct link between the tRNA-binding site and movements of the head of the small ribosomal subunit (Figure 1A) [8]. The conditional TetR knockdown method used herein was robust, resulting in diminished specific *rps13* mRNA and RPS13 protein synthesis in *T. gondii* and arrest of proliferation. This validated RPS13, and molecules associated with its regulation, as molecular targets, and the utility of this conditional ATc repressor knockdown system for such target validation. However, since RPS13 is so similar in sequence and deduced structure to other RPS13s, including human RPS13 (Figure 1B), our modeling did not offer obvious insights into targeting *T. gondii* RPS13 for medicine discovery.

Following reduction in *rps13* mRNA in the conditional knockdown parasite, western blot and immunofluorescence assays we observed that both RPS13 and RPL22 diminish but RPS13 diminishes earlier (Figure 4A,B). Diminution of RPS13 begins modestly at 4 hours and is substantial by 24 hours. RPL22 is not appreciably modulated at 4 hours, is moderately diminished by 24 hours and decreases more over the next days in culture. The ratio of 18S:28S RNA diminishes (Figure 7). This diminution of 18S rRNA indicates a role for RPS13 in either stabilization or processing of rRNA.

Conditional knockdown led to increased expression of BAG1 (Figure 6), arrest in the G1 phase of the cell cycle (Figure 7) and thereby arrest of parasite proliferation and persistent parasites in this tissue culture model. These observations suggested that depletion of ribosomes leads to synthesis of BAG1 (Figure 6) and initiates an early stress response that induces parasite G1 arrest *in vitro* (Figure 7, Table S2).

The forced proliferation arrest observed herein shares the G1 arrest feature associated with the *in vitro* pH or compound 1 stressed parasite stage. This type of G1 arrest also was observed by Radke in bradyzoites isolated from mouse brain in earlier studies [61]. However, morphology and other features of this mutant are not those of typical fully differentiated bradyzoites (Figure 6B-D). For example, Dolichos staining is absent (Figure 6). Expression data of 144 ribosomal proteins in type I-II-III tachyzoites and bradyzoites samples (expression data with the *T. gondii* Affymetrix Array available at GSE16037 and ToxoDB.org) suggests that most ribosomal proteins, including RPS13, do not have altered expression across strain or developmental stages. There are a few exceptions, however. For example, RPL4 transcript levels are increased in type III CTG. RPL4 (42.m05824) was recently found to be a part of the channel in ribosomes for assembling proteins [62] as well as being critical in stress responses in yeast [62]. Location of certain relevant RPs in ribosomes is shown in Figure 1A.

Our work provides novel insights into significance of this protein for this parasite's cell cycle, proliferation, and persistence in tissue culture after removal of ATc. Our *Δrps13* mutant parasites can persist *in vitro* in this proliferation arrested state for prolonged periods of time (months) and yet be revived by adding ATc. This is reminiscent of how plants can “go to sleep for a long, long time” through the PYR abscisic acid calcineurin stress response pathway [56]. *T. gondii* has this pathway. The recent work of Sibley et al demonstrates that this ABA system is operative in *T. gondii.*

Some of the genes that are expressed in fully differentiated bradyzoites were not transcriptionally upregulated -ATc (Table S2). This suggests that *Δrps13* can maintain functions vital for persistence while neither replicating nor differentiating to encysted bradyzoites fully. Lack of ribosomes themselves are a model leading to arrest in G1 in other organisms [3] and may also function in this manner in *T. gondii* (Figure 8). The mechanisms demonstrated *in vitro* herein suggest that stress may be translated through diminished transcription of ribosomal proteins to depletion or markedly diminished numbers of ribosomes and then to G1 arrest, at least in some cases. With an additional mechanism(s) that allows escape from the immune response, an imbalance of ribosomal proteins and diminution of ribosomes might allow a variety of microorganisms that can stop replicating and persist in a basal state to persist *in vivo* for a long time.

Our conditional mutant parasites do not appear able to evade the immune response *in vivo*. They appear to be incapable of persistence in the brain in the presence of a competent immune response. They cannot be demonstrated to be revived *in vivo* by subsequent administration of ATc administered alone or with L-NAME. Nonetheless, they protect mice against challenge with wildtype clonal Type 1 parasites completely, or clonal Type 2 parasites robustly. They are proving useful as internal “gold standard of protection” controls in ongoing studies to develop biomarkers and to produce a component vaccine to protect humans against toxoplasmosis (Cong H, McLeod R et al, in preparation).

Since bradyzoites *in vivo*, like *Δrps13 in vitro*, also are arrested in G1 [61], it seems reasonable to speculate that this diminished replication phenotype is a critical part of a response to stresses *in vivo* and could contribute to the slowed replication in the full encysted, bradyzoite phenotype. However, although it may be a component part of development of a bradyzoite phenotype and even necessary, the studies herein demonstrate that it is not sufficient for the full phenotype. At a minimum, other genes which allow for the sequestration from the host's immune response and for other essential bradyzoite genes to be expressed must also be necessary for bradyzoites to form and encyst since *Δrps13* does not persist in brain *in vivo* in the presence of a competent immune response.

It would be of interest to determine whether a similar *in vitro* persistent, G1 arrested phenotype in a “stressed” parasite is common to other parasites that are attenuated and proving to be immunogenic and protective. Examples of such parasites are those attenuated by knockout of genes encoding components of nutrient pathways and other unknown means such as in temperature sensitive mutant 4. The robustness of protection conferred by *Δrps13* combined with its lack of persistence *in vivo*, and inability to induce growth of the parasite with ATc or L-NAME after the first week, may provide insights into establishing protection by other means. These results suggest that a similar persistent exposure to immunogenic epitopes for several days might be useful in development of component vaccines as well. This conditional knockdown parasite appears to be sufficient to stimulate the immune system without establishing a chronic infection (Figure 9). Therefore it may be useful for vaccine development, including definition of epitopes and immune responses essential for protection.

### Conclusions and Significance

The studies herein address regulation of transcription of a RP and characterize effects of conditional knockdown of this RP. Promoter analysis included identification of both a transcription factor binding site in the promoter of RPS13 and associated molecules. Modulation of the transcription factors that regulate RPs would be expected to lead to subsequent imbalance of RPs. If the regulatory mechanisms present in yeast and mammals also are used by *T. gondii*, disruption of critical RPs would lead to abrogation of effect of an MDM2-like molecule, arrest in the cell cycle in G1, and hence contribute to diminution of proliferation and persistence. The work described herein, identifies mechanisms and suggests molecular switches associated with proliferation and persistence of *T. gondii in vitro*. Conditional knockdown of *rps13* which disrupts ribosomes is associated with arrest in G1 of the cell cycle. The molecular pathways in this process are molecular targets for development of antimicrobial agents to eradicate *T. gondii*. The method developed herein for conditional knockdown can be used for validation of other targets. Immunization with *Δrps13* parasites protects mice completely against subsequent challenge with wildtype clonal Type 1 parasites, and robustly protects mice against wildtype clonal Type 2 parasites. Thus, the conditional knockdown parasites have potential to be useful in vaccine development.

## Methods

### Ethics Statement

All animal studies were approved by the University of Chicago IACUC (#71734) and conducted according to AAALAC and USDA guidelines (Animal Welfare Assurance #A3523-01).

Methods are presented in detail in Text S1. They are as described in the following references: for modeling of RPS13 promoter analysis and EMSAs [63], and mass spectrometry [23], [75]–[78], ChIP [29], [52], creation of RPL22 construct [44], [45], determining expression of RPs in tachyzoite and pH switch conditions [22], homologous recombination [64], creation of constructs with four TetOs in an optimal site in the RPS13 promoter and YFP-TetR constructs [20], [21], culture of parasites [65], [66], creation of stable transfectants with the YFP-TetR [20], [21], qPCR [67], western blots, IFA [68], tissue culture of *T. gondii* and assays that measure uracil uptake [65], [66], analysis of G1 arrest [22], [46], [47], [69], transcriptomes [22] use of L-NAME [48], and immunization and evaluations of immunized mice [70].

## Supporting Information

Figure S1 (A) TRP1 and TRP2 EMSA with radio labeled TRP1 and TRP2 probes with (+) and without (−) cold competitor TRP1 or TRP2 with tachyzoite nuclear extract. Note mobility-shift which indicates that there is protein in the nuclear extract that binds to TRP2, which is competed by cold TRP2. TRP2 arrow indicates level in the gel where this occurs. (B) EMSAs with radio labeled TRP1 [left lanes] and TRP2 [right lanes] with nuclear extracts from tachyzoites competed by cold probe second lane, and with antibody to MYST A [third lane] and MYST B [fourth lane]. Some of the differences are highlighted by inclusion in green rectangles. Bands are marked by letters a to g so they can be identified in each of the corresponding lanes. In S1A * indicates a nonspecific band that is not competed by cold probe. S1A shows part of the EMSA beginning with band A. In S1A and S1B, bands of similar mobility for TRP1 and TRP2 are not necessarily binding the same nuclear extract proteins. Bands are indicated with letters to indicate corresponding bands between figures 1A, S1A, and S1B in the individual TRP1 and TRP2 elements. All EMSA results shown were reproducible in at least two replicate experiments.
(2.62 MB TIF)

Figure S2 Creation of conditional mutant parasite and replication *in vitro* +ATc. (A) Creation of parasites. These parasites were created as described previously for parasites with one TetO in the *rps13* promoter [21]. As shown, to create the *Δrps13* conditional knockdown, genomic integration of four TetO elements in the *rps13* locus was accomplished with a hit-and-run mutagenesis strategy [21], [64]. Alternative possible cross-over events could have occurred between a construct containing in sequence (TCCCCGACAACACCTTCTAC) and native *T. gondii* genomic DNA resulting in different pseudodiploid conformations [20], [21]. The constructs were previously described [21]. Pseudodiploid generation occurred creating a pseudodiploid parasite, as demonstrated by the colored diagram organization [21] with primer locations marked. Sequences from the construct in the diagram are indicated within brackets. The diagram, top, is adapted from Figure 2A in reference 21 with permission. Other symbols in this diagram include: orange box with an X represents four TetOs; blue line represents bluescript vector backbone; prps13 represents RPS13 promoter; pDHFR represents DHFR promoter; DHFR is the DHFR coding region; thick arrows represent continuation of rps13 gene. Crossovers with the construct and native gene occurred within the *rps13* promoter (prps13) as shown in the schematic diagram top, right. An alternate pseudodiploid that did not occur could have formed with a crossover within the *rps13* intron. PCR using primers 3 (GTCGAGTCCTGTAGGTTCATC) and 10 (GGAGATCTCTATCACTGATAGGGA) on DNA isolated from mycophenolic acid-xanthine-resistant clones showed that only one clone, B28, had the replacement construct integrated at the *rps13* locus (Figure S2A top, left). PCR with primers 3 and 10 yielded a product of the correct size to include the *rps13* gene promoter and four TetOs. Primers 9 (TCCCTATCAGTATAGAGATCTCC) and 4 did not amplify products (data not shown). PCR with DNA from 6-thioxanthine-resistant clones using both HXGPRT primers (Figure S2A middle) as well as primers 3 and 4 (CTCCGAAGGAGTCTCTCAGTG) (Figure S2A bottom left panel) show that pseudodiploidy has been lost for clones 1, 6, 12, 15, and 18. PCR using primers 3 and 10 (Figure S2A bottom right panel) show that clones 1, 6, 12, 15, and 18 retained TetO elements in the rps13 promoter. Parasites, e.g., chloramphenicol-resistant clones 3 and 9, express YFP (data not shown). (B) Comparison of uracil uptake in *Δrps13* mutant parasites and parental strains +ATc. Uracil uptake in the mutant parasite compared to wildtype or parental strain was similar. There was no diminution of uptake in Δrps+ATc. Parental and conditional mutant knockdown parasites are shown.
(0.61 MB TIF)

Figure S3 Expression of RPS13 and RPL22. (A) Western blot of cultures ±ATc at 4 and 48 hours probed with αRPS13 and αSAG1. (B) IFA at 24 hours and 4 days probed of cultures ±ATc with αMyc to detect Myc-tagged RPL22.
(2.22 MB TIF)

Table S1 Mass Spectrometry.
(0.07 MB XLS)

Table S2 ±ATc Transcriptome.
(0.29 MB XLS)

Text S1 (0.22 MB DOC)

Movie S1 RPS13.
(3.65 MB MOV)
