# Supplementary figures and images for: T. gondii RP Promoters & Knockdown Reveal Molecular Pathways Associated with Proliferation and Cell-Cycle Arrest
Source: PLoS One. 2010 Nov 22;5(11):e14057. doi: 10.1371/journal.pone.0014057 (PMC2989910; doi:10.1371/journal.pone.0014057)

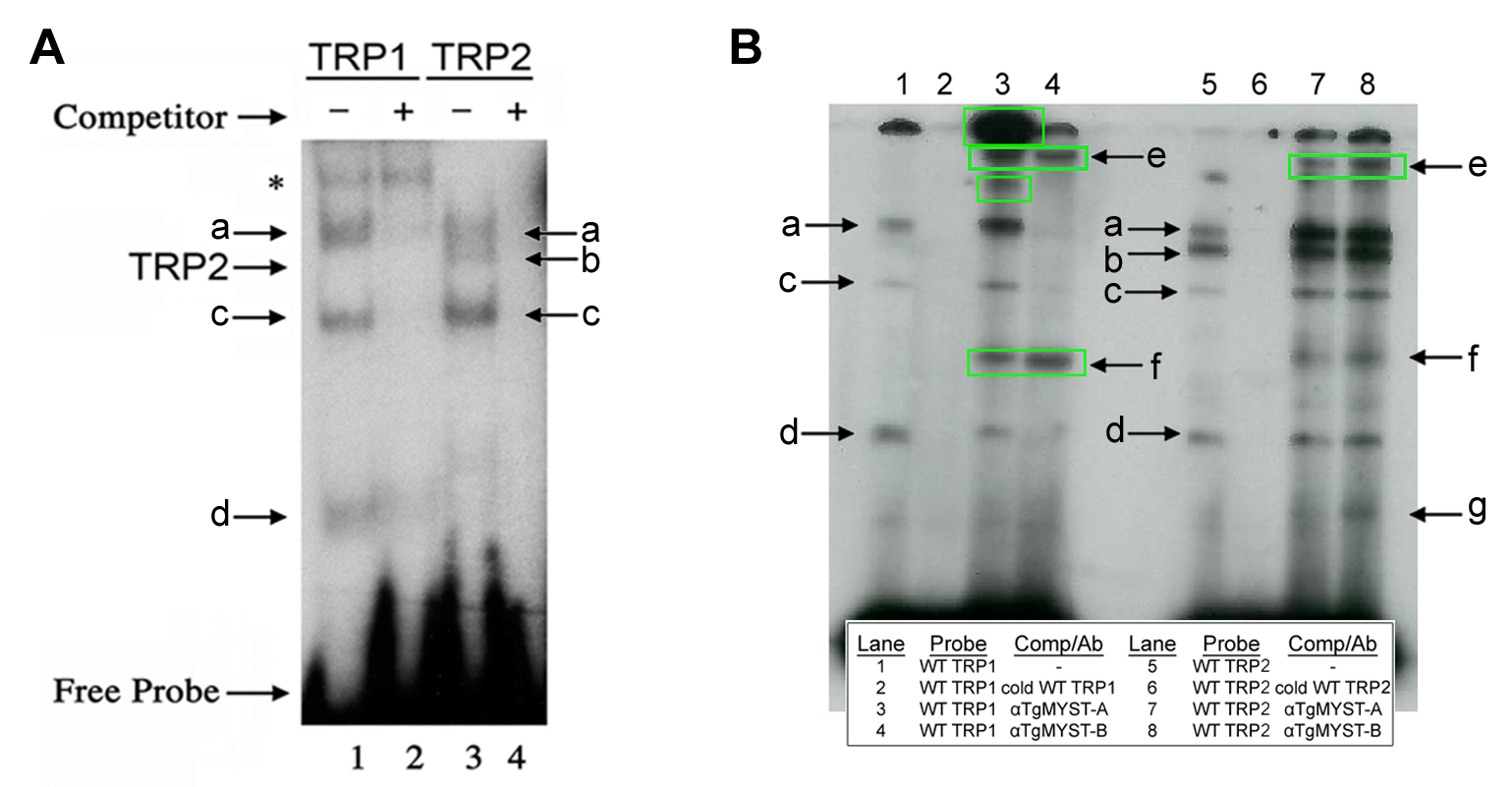

Supplement: Figure S1 — (A) TRP1 and TRP2 EMSA with radio labeled TRP1 and TRP2 probes with (+) and without (−) cold competitor TRP1 or TRP2 with tachyzoite nuclear extract. Note mobility-shift which indicates that there is protein in the nuclear extract that binds to TRP2, which is competed by cold TRP2. TRP2 arrow indicates level in the gel where this occurs. (B) EMSAs with radio labeled TRP1 [left lanes] and TRP2 [right lanes] with nuclear extracts from tachyzoites competed by cold probe second lane, and with antibody to MYST A [third lane] and MYST B [fourth lane]. Some of the differences are highlighted by inclusion in green rectangles. Bands are marked by letters a to g so they can be identified in each of the corresponding lanes. In S1A * indicates a nonspecific band that is not competed by cold probe. S1A shows part of the EMSA beginning with band A. In S1A and S1B, bands of similar mobility for TRP1 and TRP2 are not necessarily binding the same nuclear extract proteins. Bands are indicated with letters to indicate corresponding bands between figures 1A, S1A, and S1B in the individual TRP1 and TRP2 elements. All EMSA results shown were reproducible in at least two replicate experiments. (2.62 MB TIF) [file pone.0014057.s001.tif]

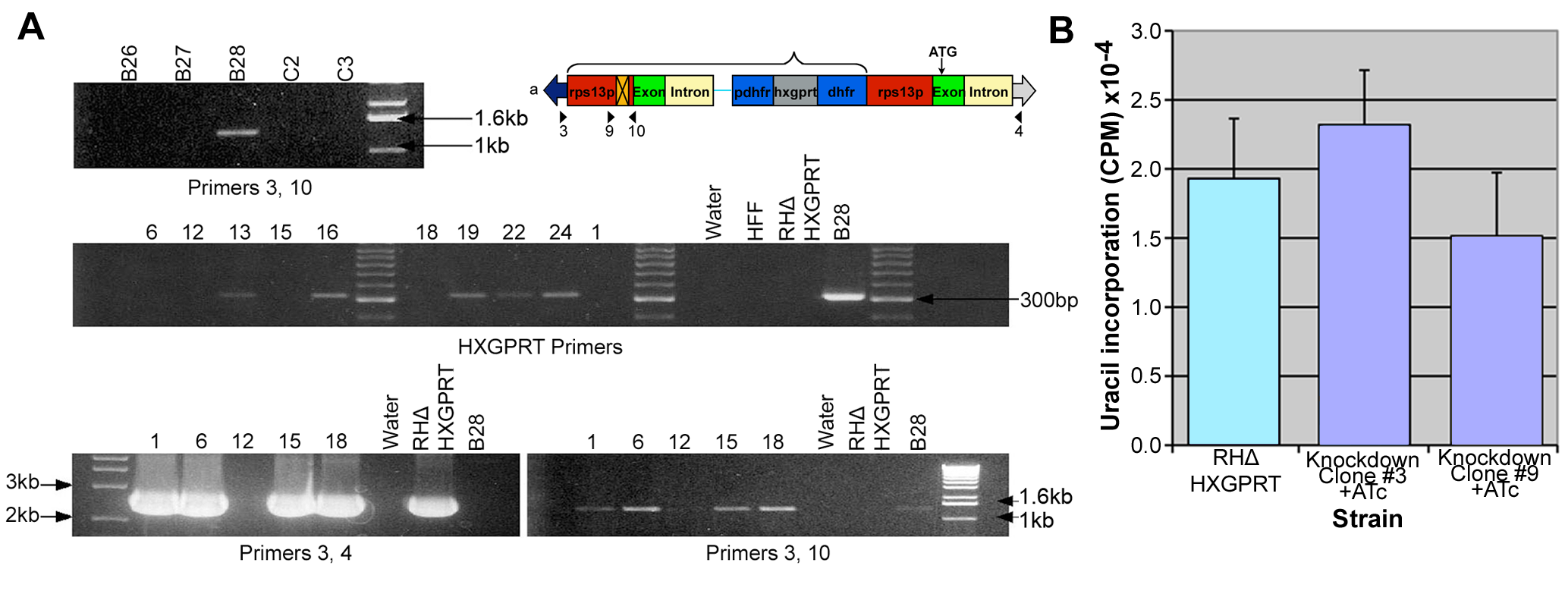

Supplement: Figure S2 — Creation of conditional mutant parasite and replication in vitro +ATc. (A) Creation of parasites. These parasites were created as described previously for parasites with one TetO in the rps13 promoter [21]. As shown, to create the Δrps13 conditional knockdown, genomic integration of four TetO elements in the rps13 locus was accomplished with a hit-and-run mutagenesis strategy [21], [64]. Alternative possible cross-over events could have occurred between a construct containing in sequence (TCCCCGACAACACCTTCTAC) and native T. gondii genomic DNA resulting in different pseudodiploid conformations [20], [21]. The constructs were previously described [21]. Pseudodiploid generation occurred creating a pseudodiploid parasite, as demonstrated by the colored diagram organization [21] with primer locations marked. Sequences from the construct in the diagram are indicated within brackets. The diagram, top, is adapted from Figure 2A in reference 21 with permission. Other symbols in this diagram include: orange box with an X represents four TetOs; blue line represents bluescript vector backbone; prps13 represents RPS13 promoter; pDHFR represents DHFR promoter; DHFR is the DHFR coding region; thick arrows represent continuation of rps13 gene. Crossovers with the construct and native gene occurred within the rps13 promoter (prps13) as shown in the schematic diagram top, right. An alternate pseudodiploid that did not occur could have formed with a crossover within the rps13 intron. PCR using primers 3 (GTCGAGTCCTGTAGGTTCATC) and 10 (GGAGATCTCTATCACTGATAGGGA) on DNA isolated from mycophenolic acid-xanthine-resistant clones showed that only one clone, B28, had the replacement construct integrated at the rps13 locus (Figure S2A top, left). PCR with primers 3 and 10 yielded a product of the correct size to include the rps13 gene promoter and four TetOs. Primers 9 (TCCCTATCAGTATAGAGATCTCC) and 4 did not amplify products (data not shown). PCR with DNA from 6-thioxanthine-resistant clones [file pone.0014057.s002.tif]

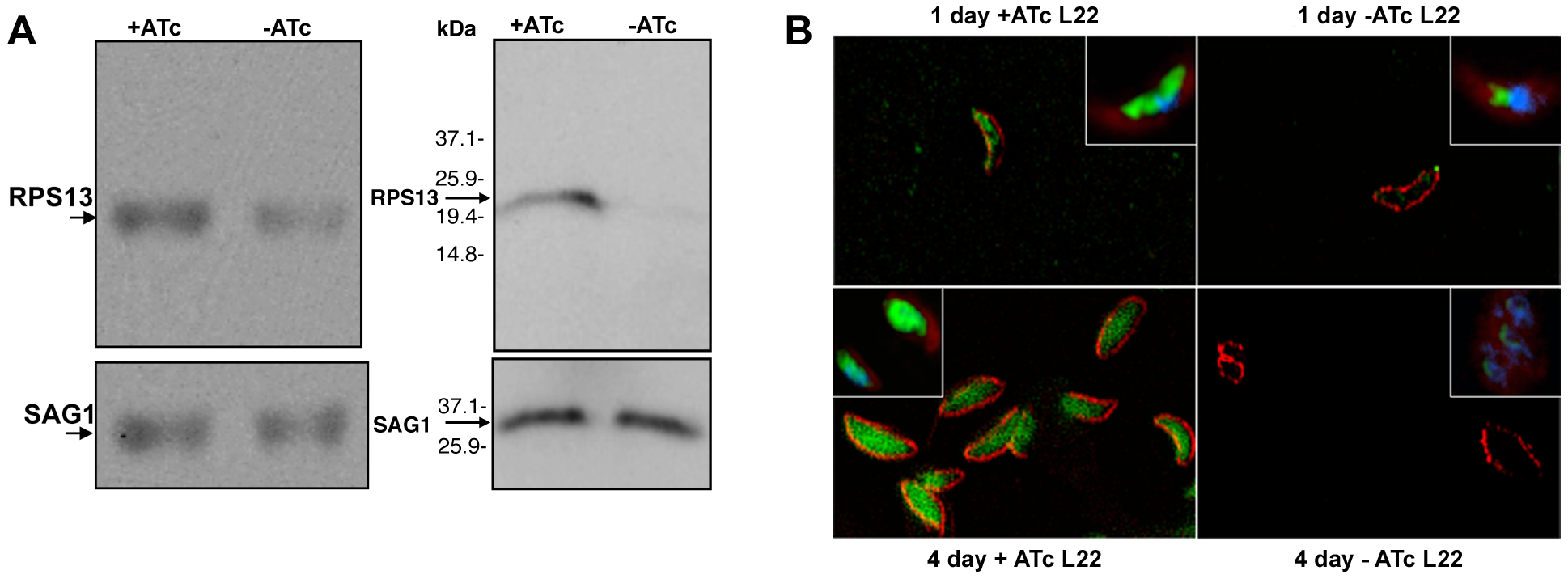

Supplement: Figure S3 — Expression of RPS13 and RPL22. (A) Western blot of cultures ±ATc at 4 and 48 hours probed with αRPS13 and αSAG1. (B) IFA at 24 hours and 4 days probed of cultures ±ATc with αMyc to detect Myc-tagged RPL22. (2.22 MB TIF) [file pone.0014057.s003.tif]
