# Supplementary material for: T. gondii RP Promoters & Knockdown Reveal Molecular Pathways Associated with Proliferation and Cell-Cycle Arrest
Source: PLoS One. 2010 Nov 22;5(11):e14057. doi: 10.1371/journal.pone.0014057 (PMC2989910; doi:10.1371/journal.pone.0014057)
Supplement: Text S1 — (0.22 MB DOC) [file pone.0014057.s006.doc]

***T. gondii RP Promoters & Knockdown Reveal Molecular Pathways Associated with Proliferation and Cell-cycle Arrest***

Samuel L. Hutson^1^, Ernest Mui^1^, Karen Kinsley^1^, William H. Witola^1+^, Michael S. Behnke^2#^, Kamal El Bissati^1^, Stephen P. Muench^3^, Brittany Rohrman^1†^, Susan R. Liu^1^, Robert Wollmann^1^, Yuko Ogata^4^, Ali Sarkeshik^5^, John R. Yates III^5^, and Rima McLeod^1*^

^1^Departments of Surgery (Ophthalmology) [SLH, EM, KK, WHW, BR, SRL, RM) and Pediatrics (Infectious Disease) (RM), Committees on Immunology, Molecular Medicine, and Genetics, Institute of Genomics and Systems Biology, and The College (RM), and Pathology (RW), The University of Chicago, Chicago, Illinois, USA

# ^2^Department of Veterinary Molecular Biology, Montana State University, Bozeman, Montana, USA

^3^Institute of Membrane and Systems Biology, University of Leeds, Leeds, United Kingdom

^4^Seattle Biomedical Research Institute, Seattle, Washington, USA

^5^Department of Chemical Physiology, Scripps Research Institute, La Jolla, California, USA

^#^Current Address: Department of Molecular Microbiology, Washington University School of Medicine, St. Louis, Missouri, USA

^+^Current Address: Department of Zoonotic Diseases and Veterinary Medicine, Tuskegee University, Tuskegee, Alabama, USA.

^†^Current Address: Department of Bioengineering, Rice University, Houston, Texas, USA

^*^Please address correspondence to:

Rima McLeod, M.D.

The University of Chicago

Room AMBH S206

5841 South Maryland Avenue

Chicago, Illinois 60637

Phone: 773-834-4130

Fax: 773-834-3577

Email: rmcleod@midway.uchicago.edu

**1. Methods**

***The following are the detailed methods for the supplemental data:***

***Modeling Structures of RPS13:***

The phyre server (http://www.sbg.bio.ic.ac.uk/3dpssm/) was used to obtain a model for the *T. gondii* RPS13 structure based on the related *T. thermophilus* RPS15 structure that shares significant sequence similarity to the RPS13 family. A sequence alignment was made with various RPS13 enzymes (human, malarial, bacterial) and the RP15 protein from *Thermophilus* for which the *T. gondii* RPS13 structure is based. Residues fully conserved and highly similar were identified as defined by ClustalW. Similarities in residues which interact with ribosomal RNA were noted. A movie showing the similarities and these features of RPS15 and RPS13 are available in supplementary material.

# *Promoter analyses*

***TRP1 ad TRP2: Identification in the Genome and EMSA using TRP1 and 2 promoter elements with mutational analyses***

Promoters of genes encoding ribosomal proteins contain highly conserved binding sites called TRP elements [20,21]. Consensus sequences of these TRP elements, TRP1 (TCGGCTTATATTCGG) and TRP2 ([T/C]GCATGC[G/A]), are located close to transcriptional start sites. These were selected because van Poppel and Schaap [19-21] noted that in yeast, activating and repressive elements for ribosomal protein transcription and growth or differentiation under starvation conditions have been identified and elements have been shown to play a role in transcription activation. To determine the binding occupancy of the TRP elements in *T. gondii*, gel electrophoresis mobility-shift assays using nuclear extracts isolated from tachyzoites were performed using these two promoter elements, TRP1 and TRP2, postulated to regulate expression of ribosomal proteins, one as an activator of transcription and the second as a suppressor of transcription, EMSA was used to identify the location in the gel with mobility-shift where proteins in *T. gondii* lysates from tachyzoites bind to the TRP elements, similar to those that regulate activators and suppressor of transcription in yeast.

EMSA were performed using *T. gondii* tachyzoite nuclear extracts with TRP1 and TRP2. Nuclear extracts were made from *T. gondii* as previously described [63]. Nuclear extracts were obtained from *T. gondii* RH tachyzoites as follows: 2 x 10^8^ parasites were harvested by scraping infected fibroblasts from flasks, passing this twice through a 25g needle, isolating parasites using a 3μ filter, and then washing once in cold PBS, pH 7.4 by centrifugation at 1,500g for 10 minutes. They were then resuspended in 1.6ml of ice-cold buffer A (10mM HEPES pH 7.9, 1.5mM MgCl2, 10mM KCl, 0.5mM DTT, 0.1mM EDTA, 0.65% NP40 and 0.5mM PMSF). Parasite lysate was incubated for 10 minutes on ice and then centrifuged at 1,500g for 10 minutes at 4°C. Supernatant was discarded and pellet containing nuclei was lysed by resuspending in 160μl of Buffer B (20mM HEPES pH 7.9, 1.5mM MgCl2, 420mM NaCl, 0.2mM EDTA, 0.5mM DTT, 25% glycerol and 0.2mM PMSF). After incubation on ice for 15 minutes, nuclear extracts were centrifuged at 14,000g for 10 minutes at 4°C. Supernatant was aliquoted in 30ml volumes and stored at -80°C. Protein concentration of the nuclear lysate was determined by Bradford Method. Synthetic oligonucleotides containing *Toxoplasma* ribosomal protein (TRP) motifs derived from *rps13* promoter 1 (5’ GCGCGTCCTCGGCTTATATTCGGAAAAG 3’) and 2 (5’ GCGAACGTGATAATGCATGAACTCGAT 3’) were annealed to their complimentary oligonucleotides (5’ GCGTTCCTTTTCCGA ATATAAGCCGAGG 3’ and 5’ GCGATGATCGAGTTCATGCATTATCAC) and labeled with ^32^P-phosphate end labeling (dCTP by Klenow fragment).

**TRP1 Upper Strand:** (**From RPL12**)

5’ CGTCCTCGGCTTATATTCGGAAAAG 3’ (sequence copied from TRP1 L12)

GCG inserted at 5’

Final TRP1 Upper Strand: 5’ GCGCGTCCTCGGCTTATATTCGGAAAAG 3’ (28bp)

**TRP1 Lower Strand:**

5’ cctcggcttatattcggaaaaggaa 3’ (sequence copied from TRP1 L12)

3’ GGAGCCGAATATAAGCCTTTTCCTT 5’ (complementary sequence)

5’ TTCCTTTTCCGAATATAAGCCGAGG 3’ (reverse complementary sequence)

GCG inserted at 5’

Final TRP1 Upper Strand: 5’ GCGTTCCTTTTCCGAATATAAGCCGAGG 3’ (28bp)

**TRP2 Upper Strand:** (**From RPS13)**

5’ AACGTGATAATGCATGAACTCGAT 3’ (sequence copied from rps13 TRP2)

GCG inserted at 5’

Final TRP2 Upper Strand: 5’ GCGAACGTGATAATGCATGAACTCGAT 3’ (27bp)

**TRP2 Lower Strand:**

5’ GTGATAATGCATGAACTCGATCAT 3’ (sequence copied form rps13 TRP2)

3’ CACTATTACGTACTTGAGCTAGTA 5’ (complementary sequence)

5’ ATGATCGAGTTCATGCATTATCAC 3’ (reverse complementary sequence)

GCG inserted at 5’

Final TRP2 Lower Strand: 5’ GCGATGATCGAGTTCATGCATTATCAC 3’ (27bp)

***Final oligonucleotides from EMSAs were derived as follows:***

Flanking sequences for TRP1 are in *rpl12* and for TRP2 in *rps13* DNA binding reactions were carried out with 20 x 10^3^ cpm of probe and 10μg of nuclear extract in a buffer containing 50mM Tris pH 7.5, 200mM NaCl, 50mM DTT, 5mM EDTA pH 8.0 in the presence of poly d(I-C) 1μg per reaction. Binding reactions were incubated for 20 minutes at room temperature and electrophoresed on a 5% native polyacrylamide gel using 0.25X TBE. Antibody supershift assays were performed by incubating the binding reaction with anti-MYST antiserum 10 minutes prior to probe addition.

***Linker scan mutational analysis***

In order to determine the specific nucleotide sequences that are critical for the TRP2 complex, 3 bp linker scan mutational analysis across the DNA binding sequences was carried out. Using these mutant oligos, EMSAs were carried out with nuclear extract as described previously [30]. The mutant oligos that failed to generate bound complex would indicate precise sequences required for binding.

***Analysis of nuclear extract proteins associated with TRP1 and TRP2*.**

***Preparation of Samples for Mass Spectroscopy***

Electrophoretic mobility shift assays (EMSA) were performed to characterize the complex visualized in the initial assays. The shifted bands obtained in EMSA were excised, destained and subjected to in-gel trypsin digestion. Trypsin digestion of dried gel pieces was performed overnight at 37 °C with 5 ng/µL trypsin (Promega Corporation, Madison, WI) in 50 mM ammonium bicarbonate. The peptides were extracted using 5% v/v formic acid in water after 30 min incubation, then with 5% formic acid in 50-60% v/v acetonitrile. The pooled extracts were concentrated to less than 5 µL *in vacuo*, and stored at -20 °C.

***LC-MS/MS analysis.***

LC-MS/MS was performed using LTQ linear ion trap mass spectrometer (Thermo Fisher Scientific). The peptide samples were loaded onto the reversed phase column using a two-mobile-phase solvent system consisting of 0.4% acetic acid in water (A) and 0.4% acetic acid in acetonitrile (B). The mass spectrometer operated in a data-dependent MS/MS mode over the *m/z* range of 400-2000. For each cycle, the five most abundant ions from each MS scan were selected for MS/MS analysis using 45% normalized collision energy. Dynamic exclusion was used to exclude ions that had been detected twice in a 30 sec window for 3 min. Raw MS/MS data were submitted to Bioworks 3.3 (ThermoElectron, San Jose, CA, USA) and searched using the Sequest algorithm against T. gondii protein database v. 5.2 (http://toxodb.org/toxo/), which included additional common contaminants i.e. human keratin. The Sequest output files were analyzed and validated by PeptideProphet [75]. Proteins and peptides with a probability score of >0.9 were accepted. In an initial experiment Raw Xtract Software version 1.9.9 (78) a DTASelect version 2.0.37h (76, 77) were used and values ranging from >0.75 to >0.9 for DTASelect were accepted.

***Chromatin immunoprecipitation (ChIP)***

ChIP was performed on RH strain tachyzoites stably expressing a Flag-tagged form of recombinant TgMYST B. ChIP assay was performed as previously described [29, Figure 2], using polyclonal anti-Flag antibody (Sigma F7425) immobilized to Dynabeads Protein A (Invitrogen). Primers used in the PCR of immunoprecipitated DNA are:

RPS13 – 1 sense: 5’-TGCCTTTTCCTGGGGCACACTGCA

RPS13 – 1 antisense: 5’-AAATTGCATGCGCCCCCGTGGCTCG

RPS13 – 2 sense: 5’-ACGAGTGCGTCTCTTGCATCCGAG

RPS12 – 2 antisense: 5’-ACGCTCGCGACAAAAGCCCCCA

L12 – 1 sense: 5’-TCATGCCGAAGTCAGCTTGGAT

L12 – 1 antisense: 5’-AAGCGCTTTCGACCGACAGAA

L12 – 2 sense: 5’-AACCATCGTCTGCGAGAGACA

L12 – 2 antisense: 5’-CAGCCTTCAATGCATGCGCACAA

An identical amount of the same input DNA was utilized both with and without αMYST B. In our CHiP studies which suggest there may be associations of MYST B and TRPs, the “No antibody” control used is the standard control and there were no bands in the ChIP (Figure 1). Recent experiments [52] provide additional information demonstrating specificity of the same reagents and methodology used herein. Separate studies for another purpose [52] were performed with the same antibody to the FLAG tag, the same parasites in which MYST B is flag tagged, and the identical methodology was used in the same laboratory. In these studies, a region of the genome (i.e., in the actin gene) where the protein of interest should not be present, an unrelated antibody also was included as negative controls [52]. This further demonstrated specificity of this antibody. Details of methods for PCR and ChIP are the same as described in [52]. ChIP was performed on tachyzoites stably expressing fMYST-B using polyclonal anti-FLAG antibody (Sigma F7425) immobilized to Dynabeads Protein A (Invitrogen). Immunoprecipitated DNA samples were normalized with a standard curve using serially diluted input DNA. 0.1 ng of total ChIP DNA was used in each reaction with reactions performed in triplicate. It was noted in parallel studies that actin mRNA was not increased in fTgMYST-B over-expressing parasites using these methods and reagents [52].

# *Creation and characterization of Δrps13:*

# *Cell and Parasite Culture*

*T. gondii* tachyzoites were cultured at 37°C and 5% CO_2_ in human foreskin fibroblasts (HFF) grown in Iscove’s Modified Dulbecco’s Medium (IMDM) supplemented with 10% fetal bovine serum, 100 I.U. ampicillin, 100μg/mL streptomycin, 25ng/mL amphotericin B and 2mM of either L-glutamine or GlutaMAX™-1 (Invitrogen) [65,66]. RPS13-conditional knockdown clones were maintained in 20μM chloramphenicol and 100ng/mL anhydrotetracycline. Wildtype RH and RHΔHXGPRT strains were also cultured.

***Plasmid constructs and generation of mutant parasites for conditional knockdown of RPS13***

Plasmid pDHFR*HXGPRT*/*rps13*subTetO(IV)-23 bearing a fragment of approximately 1250 bp of RPS13 promoter and 1250 bp of RPS13 UTR plus the coding sequence in which four TetO elements had been integrated in tandem just upstream of the transcription start site was generated by Hit-and-run site directed mutagenesis [20,21,64]. This plasmid was created and provided for use herein by Dick Schaap, Nicole Van Poppel, Jelle Welagen at Intervet. Fifty μg of pDHFR*HXGPRT*/*rps13*subTetO(IV)-23 plasmid DNA was electroporated into 10^7^ RHΔHXGPRT tachyzoites in 400μL of cytomix (120mM KCl, 150μM CaCl_2_, 10mM K_2_HPO_4_/KH_2_PO_4_-pH 7.6, 25mM HEPES-pH 7.6, 2mM EDTA and 5mM MgCl_2_, freshly supplemented with 2mM ATP and 5mM glutathione) at 1.5 kV and 25μF. After 15 minutes of incubation at room temperature, all parasites were used to infect a 75 cm^2^ confluent flask of HFF. After 4 hours incubation at 37°C, the medium was replaced with fresh medium and parasites were left overnight at 37°C, after which the medium was supplemented with 25μg/mL mycophenolic acid and 50μg/mL xanthine to select for the presence of the HXGPRT gene [64]. Parasites were passed at 1 to 2 day intervals for 10 days under this selection and resistant clones were generated by limited dilution. Fifty-one HXGPRT-positive clones were obtained, and pseudodiploidy was assessed by PCR using either primers 3 and 10 or primers 4 and 9 (Figure 2). Only 1 out of 51 clones was a pseudodiploid, clone B28, and this clone was a Type 1 pseudodiploid as primers 3 and 10 amplified the appropriate sized band (Figure 2A top). No bands were detected in any clones using primers 4 and 10 (data not shown). Clone B28 was passed without drug selection pressure to allow spontaneous intrachromosomal recombination between the two *rps13* loci, after which the HXGPRT gene was negatively selected by culturing in 6-thioxanthine. Ten 6-thioxanthine-resistant clones were obtained, and PCR using HXGPRT-specific primers was used to determine whether the recombination had occurred (Figure 2A middle). Five out of 10 clones, clones 1, 6, 12, 15 and 18, were HXGPRT-negative. Since this intrachromosomal recombination could yield parasites with a TetO-modified promoter or a wildtype promoter, PCR with primers 3 and 10 was used to confirm TetO retention, and PCR using primers 3 and 4 was used to confirm pseudodiploid resolution (Figure 2A bottom). Of these, clones 1, 6, 15 and 18 clearly had the *rps13* promoter replaced, while the genomic identity of clone 12 is uncertain.

***Transfection of mutant parasites with the TetR expressing plasmid***

A construct containing the gene encoding a yellow fluorescent-Tet-repressor (YFP-TetR) chimeric protein was created as described previously [20,21] and used in accordance with MTA #4 from Intervet. This plasmid confers chloramphenicol resistance using a chloramphenicol acetyltransferase (CAT) cassette. Fifty μg of the YFP-TetR construct [20,21] was electroporated into TetO-containing clone #1 as described above. Tet repressor-containing parasites were selected for by passing as needed for 2 weeks in 20μM chloramphenicol and 100ng/mL anhydrotetracycline, after which clones were generated by limited dilution.

# **Selection of YFP-TetR expressing mutant parasites by Fluorescence Microscopy**

# This was performed as described in Van Poppel [20,21].

# **RNA Isolation and Quantitative Real-Time PCR (qRT-PCR)**

Parasites were isolated from host cells by two successive passages through a 27.5 gauge needle and one passage through a 3μ filter, followed by centrifugation at 500*g* for 10 minutes. Total RNA was isolated from cell pellets using TRIzol Reagent (Invitrogen), and mRNA was isolated from total RNA using Oligotex mRNA Isolation Kit (QIAGEN). First strand cDNA synthesis was performed using M-MLV reverse transcriptase (Promega), and this was used as template in qRT-PCR experiments. qRT-PCR was performed on an Applied Biosystems 7300 Real-Time PCR System and analyzed using the accompanying SDS Software. Small DNA fragments (approximately 500 bp) of *T. gondii* *rps13* cDNA were amplified by PCR and various amounts were used as template to generate standard curves. No template controls were performed in each experiment.

# **Primers**

| Primer | Sequence |
| --- | --- |
| 3 | 5’-gtcgagtcctgtaggttcatc-3’ |
| 4 | 5’-ctccgaaggagtctctcagtg-3’ |
| 9 | 5’-tccctatcagtatagagatctcc-3’ |
| 10 | 5’-ggagatctctatcactgataggga-3’ |
| HXGPRT 5’ | 5’-tccccgacaacaccttctac-3’ |
| HXGPRT 3’ | 5’-gattgacaagtcgtcgctca-3’ |
| RPS13 Real-Time 5’ | 5’-taaatccgtcactggcaaca-3’ 529 f9x |
| RPS13 Real-Time 3’ | 5’-tgtccttcctgtttctctcca-3’ |
| BAG1/5 5’ | 5’-ggcgttctacgtgtcacgatcaag-3’ |
| BAG1/5 3’ | 5’-catcaaccaggttttctcggctg-3’ |
| SAG1 5’ | 5’-cggttgtatgtcggtttcgctg-3’ |
| SAG1 3’ | 5’-tgttgggtgagtacgcaagagtgg-3’ |

***Western blot analysis of RPS13 production at 4 and 24 hours after removal of ATc***

Two sets of *ΔRPS13* mutant and wildtype parasites were grown in fibroblasts for 24 hours in the presence of ATc. Thereafter, the infected fibroblasts were washed three times with fresh medium -ATc. ATc was then added to one set of parasites while the other set was maintained -ATc and the parasites cultured for 4 hours or 24 hours. Parasites were isolated from host cells by two successive passages through a 27.5 gauge needle and one passage through a 3μ filter, followed by centrifugation at 500*g* for 10 minutes. The parasite pellet was washed twice in PBS and lysed in SDS sample buffer. Equal protein amounts were loaded to wells of a 12% SDS-polyacrylamide gel and fractionated by electrophoresis. The proteins were transferred to nitrocellulose membranes and immunoblotting was done using either rabbit anti-RPS13 (provided by Dick Schaap) or mouse anti-SAG1 antibodies as primary antibodies and then with conjugated goat anti-rabbit and anti-mouse as the secondary antibodies. Signal generation was performed using an ECL chemiluminescence kit (PerkinElmer Life Sciences).

***Generation and analysis of RPS13 mutant parasites expressing Myc-tagged ribosomal large subunit protein 22 (RPL22)***

To generate a plasmid construct for expression of chimeric RPL22-Myc protein under phleomycin selection pressure, the RLP22-Myc cassette was excised from the original construct (kindly provided by David Morris [44]) by HindIII digestion. The approximately 2 kb cassette contained a promoter and a terminator derived from the respective endogenous RLP22 gene of *T. gondii*. The fragment was ligated at the HindIII site of the pGRA1/ble plasmid [45] that possesses a phleomycin selection marker. The resultant recombinant plasmid was transfected into the RPS13 mutant parasites and stable transfectants were selected with 5μg/ml phleomycin (Sigma). Stable transfectants were cultured in the presence or absence of ATc for 1 or 4 days and the parasitized fibroblasts that had been grown on cover slips were washed in PBS and fixed for 30 minutes in a solution of 1% paraformaldehyde in PBS. After rinsing in PBS, the cover slips were incubated in blocking buffer (1% fetal bovine serum, 0.1% saponin in PBS) for 1 hour. The cover slips were then incubated with a mixture of mouse anti-SAG1 (1:500 dilution) and rabbit anti-Myc (1:500 dilution) antibodies for 1 hour at room temperature. Following three washes in blocking buffer, the cover slips were incubated with a mixture of anti-mouse antibody (1:500 dilution) conjugated to Texas red dye and anti-rabbit antibody (1:500 dilution) conjugated to fluorescein isothiocyanate for 1 hour at room temperature. After rinsing, the coverslips were mounted on slides with ProLong Gold Antifade reagent with DAPI Antifade (Molecular Probes, Invitrogen) and analyzed by fluorescence microscopy. The same antibodies as used to detect RPS13 in Western blots were used for FA.

***Western blot analysis of RPL22 expression at 4 and 24 hours after removal of ATc***

The same procedure utilized for the detection of RPS13 by Western blotting was performed with RPS13 mutant parasites expressing Myc-tagged RPL22, probing for both RPS13 and RPL22 in the parasites’ protein extracts using αRPS13 and αMyc antibodies described above.

***^3^H-Uracil Incorporation Assay***

Fifty *rps13* conditional knockdown or RH control parasites were used to infect individual wells of confluent HFF monolayers in 96-well plates with or without 100ng/mL anhydrotetracycline. These were incubated at 37°C for 3 days, at which point 2.5 μCi of tritium-labeled uracil was added to each well [65,66]. After 24 hours of further incubation, cells were then transferred onto filter plates using a cell harvester and uracil incorporation was measured using a liquid scintillation counter (Packard).

***IFA for BAG1, SRS9/BRS4, and Dolichos staring***

Immunofluorescence assay analysis of *T. gondii* cyst wall formation by Dolichos and expression of SAG1, BRS4/SRS9 and BAG1 protein was carried out as follows: Our *T. gondii* parasites that were genetically modified to establish an *rps13* gene knockdown system in the absence of anhydrotetracycline (ATc) as described above were utilized. The parasites were cultured in the presence or absence (+/-) of ATc for 48 hours. In control cultures, separately, for parasites +ATc, the pH of the culture medium was raised to pH8.0 in RPMI medium containing NaOH and 10 mM tris (to stress parasites and induce bradyzoites and cyst wall formation) over a period of 120 hours. These parasites from each culture condition were washed in PBS and fixed in 1% paraformaldehyde. The parasitized fibroblasts were then permeabilized with blocking buffer containing 0.2% tritonX. The parasites were then incubated with mouse α-SAG1 (1:500 dilution), after which they were washed and incubated with secondary antibody, goat α-mouse-conjugated to Texas red, and with Dolichos conjugated to FITC. After washing the cells were incubated with Hoechst stain to stain the nuclei and washed, dried and mounted in anti-fade prolong. The slides were examined by fluorescence microscopy.

***G1 analysis using FACS***

Tachyzoites expressing yfp were cultured for 24 hours in the presence or absence of ATc. Fixation and propidium iodide (PI) staining of parasites followed previously described methods [22,46,47,69]. Briefly, parasites were fixed in 70% ethanol at -20C > 24 hours, pelleted, suspended in 0.5ml of PI staining solution, 250U RNase cocktail was added, and incubated in the dark at room temperature for 30 minutes. The DNA content of 10,000 parasites was determined by flow cytometry and analyzed using CellQuest software (BD Biosciences, San Jose, CA).

***RNA purification, probe construction, microarray hybridizations and analysis.***

*T. gondii* RPS13 conditional mutant parasites were grown in the presence or absence of ATc for 24 hours in T175 flasks. Fifty million parasites from each flask were harvested by scraping and then isolated from host cells by two successive passages through a 25 gauge needle and one passage through a 3μ filter. Total RNA was processed using the RNeasy protocol (Qiagen, Valencia, CA) with mercaptoethanol added to the lysis solution and DNase-I treatment performed prior to RNA elution. RNA sample quality was assessed on a model 2100 Bioanalyzer (Agilent Biotechnologies, Foster City, CA). Synthesis and fragmentation of cRNA probes followed Affymetrix one-cycle protocols and hybridizations to the *Toxoplasma* GeneChip array (see http://ancillary.toxodb.org/docs/Array-Tutorial.html for a description of the array design) was performed on the Affymetrix GeneChip Station following standard methods as previously described [22]. All hybridization data are available at the NCBI GEO record GSE20616. To minimize variations among independent experiments, samples were processed together for probe synthesis and hybridization. For each sample type, two independent RNA samples were harvested from which two cRNA samples were created for a total of four hybridizations per sample type (two independent, two replicate). Hybridization data were preprocessed with Robust Multiarray Average (RMA) and normalized using per chip normalize to 50^th^ percentile and per gene normalize to median and analyzed using the software package GeneSpring 7.2 (Agilent Technologies, Santa Clara, CA). Genes with raw expression values in both sample types less than or equal to 25 were removed from the analysis. A Welch T-test was used to identify genes with statistically significant differences in expression; parametric test, variances not assumed equal, *P*-value cutoff 0.01, multiple testing correction: Benjamini and Hochberg False Discovery Rate. This restriction tested 4,154 genes. About 1.0% of the identified genes would be expected to pass the restriction by chance.

***In vivo Studies of rps13 and Challenges:***

# **Infection with rps13 conditional mutant followed by treatment +ATc at various times after initial infection, ±L-NAME**

Infection was with the *rps13* conditional mutant ±ATc. In the first experiments, two groups of five female Swiss Webster mice aged 6 to 10 weeks were infected with 100 *rps13* conditional knockdown clone 3 tachyzoites. One group was given 0.2mg/mL ATc in their drinking water and one group was given water. A third group of 5 mice was not infected but given ATc in water. Twenty days post-infection, surviving mice were infected with 2,000 RH tachyzoites. Additional mice, 3 infected -ATc and 3 uninfected, were kept separately as histology controls. This experiment was repeated at least twice.

Subsequently, in separate experiments, ATc also was given at the time of infection with 100,000 conditional mutant parasites or at later times, i.e., at 1, 2, 3, or 4 weeks after infection with the conditional mutant.

In addition, in separate experiments two groups of five female Swiss Webster mice aged 6 to 10 weeks were infected with 100 *rps13* conditional knockdown clone 3 tachyzoites. One group was given 0.2mg/mL ATc in their drinking water and one group was given water. A third group of 5 mice was not infected but given ATc in water. Another group was given ATc and L-NAME [48] to inhibit iNOS.

# **Infection with rps13 conditional mutant followed by challenge with RH strain T. gondii or Me49 strain of T. gondii, quantitation of cysts, brain histopathology and qPCR**

The three groups of mice described above were challenged with RH *T. gondii*. These two groups of five female Swiss Webster mice aged 6 to 10 weeks that were infected with 100 *rps13* conditional knockdown clone 3 tachyzoites, with one group given 0.2mg/mL ATc in their drinking water and one group given water. A third group of 5 mice was not infected but given ATc in water. Twenty days post-infection, surviving mice were infected with 2,000 RH tachyzoites.

In separate experiments unimmunized mice as controls and mice immunized with 1,000 or 10,000 or 100,000 *rps13* conditional mutant parasites were challenged with 1,000 tachyzoites of the RH strain of *T. gondii* 4 weeks after immunization. In additional experiments, mice immunized with 100,000 *rps13* conditional mutant parasites on three occasions, 2 weeks apart were challenged with the RH strain of *T. gondii*. Each experiment was performed at least twice.

In addition, similar challenges with Type 1 parasites were performed approximately one year after initial immunizations. Each experiment was performed twice.

Infection with *Δrps13* with one or two (two weeks apart) immunizations with 100,000 mutant tachyzoites followed by challenge with 50 cysts intraperitoneally of the Me49 strain *T. gondii*. Thirty days later the brain of challenged mice was isolated and divided into the two hemispheres sagitally. Half the brain tissue was homogenized in 0.5ml saline. The other half was placed in 10% formalin for histopathology. A 100 microliter aliquot was used to quantify cysts were quantified using microscopic preparations of the homegenate with the identity of the sample obscured from the person enumerating the cysts and with control normal brain sample included as well. The remaining homegenate was snap frozen and stored at -80°C until used for PCR. Brain histopathology was performed as described previously quantifying cyst number, intraparenchymal inflammatory process, perivascular inflammatory process in the brain parenchyma and in the leptomeninges, and especially noting if the perihippocampal and intrahippocampal perivascular process described earlier was noted. Whether the inflammatory process was focal or widely distributed also was noted. The slides were evaluated by two separate observers, neither of who were aware of the treatment of the mice from which the tissues were obtained at the time they were evaluated. Quantitative PCR to detect *T. gondii* DNA (300 copy 529 bp gene) was performed as described [67], again without knowledge of the treatment of the mice from which tissue was obtained and with internal controls.

***Statistics***

All experiments were replicated at least twice. Numbers of mice in each experiment are indicated in graphs or tables. Significance of differences were determined using Student’s T-test when 2 groups were compared.

For the cyst count and qPCR data, analysis of variance (ANOVA) was first used to compare levels across all three groups separately for each experiment and then overall, combined across experiments. For the combined analysis, experiment was included as a blocking factor in the model to account for experiment to experiment variability. If the overall p-value from the ANOVA was statistically significant, then pairwise comparisons were performed with a Bonferroni correction to account for the multiple comparisons being made. The square root transformation was used in this analysis due to non-normality. However, the summary statistics provided in the tables are based on the untransformed data. The histopathology data was analyzed similarly. A *P*-value ≤ 0.05 was considered statistically significant. Analyses were performed using Stata Version 11 (StataCorp., College Station, TX).

**2. Table S1: Mass Spectrometry**

**3. Table S2: ±ATc Transcriptome**

**4. Movie S1:** RPS13
